# The fusion of multi-omics profile and multimodal EEG data contributes to the personalized diagnostic strategy for neurocognitive disorders

**DOI:** 10.1186/s40168-023-01717-5

**Published:** 2024-01-19

**Authors:** Yan Han, Xinglin Zeng, Lin Hua, Xingping Quan, Ying Chen, Manfei Zhou, Yaochen Chuang, Yang Li, Shengpeng Wang, Xu Shen, Lai Wei, Zhen Yuan, Yonghua Zhao

**Affiliations:** 1 State Key Laboratory of Quality Research in Chinese Medicine, Institute of Chinese Medical Sciences, University of Macau, Avenida da Universidade, Taipa, 999078 Macau SAR China; 2 Centre for Cognitive and Brain Sciences, University of Macau, Avenida da Universidade, Taipa, 999078 Macau SAR China; 3 School of Health Economics and Management, Nanjing University of Chinese Medicine, Nanjing, 210023 Jiangsu China; 4 Kiang Wu Nursing College of Macau, Macau, 999078 China; 5 Department of Gastrointestinal Surgery, Second Clinical Medical College of Jinan University, Shenzhen People’s Hospital, Shenzhen, 518020 China; 6 Jiangsu Key Laboratory of Drug Target and Drug for Degenerative Diseases, Nanjing University of Chinese Medicine, Nanjing, 210023 China; 7 School of Pharmaceutical Science, Southern Medical University, Guangzhou, 510515 China; 8 Department of Pharmaceutical Sciences, Faculty of Health Sciences, University of Macau, Taipa, Macau SAR 999078, China

**Keywords:** Neurocognitive disorders, Electroencephalography, Metagenomics, Proteomics, Metabolomics, Support vector machine

## Abstract

**Background:**

The increasing prevalence of neurocognitive disorders (NCDs) in the aging population worldwide has become a significant concern due to subjectivity of evaluations and the lack of precise diagnostic methods and specific indicators. Developing personalized diagnostic strategies for NCDs has therefore become a priority.

**Results:**

Multimodal electroencephalography (EEG) data of a matched cohort of normal aging (NA) and NCDs seniors were recorded, and their faecal samples and urine exosomes were collected to identify multi-omics signatures and metabolic pathways in NCDs by integrating metagenomics, proteomics, and metabolomics analysis. Additionally, experimental verification of multi-omics signatures was carried out in aged mice using faecal microbiota transplantation (FMT). We found that NCDs seniors had low EEG power spectral density and identified specific microbiota, including *Ruminococcus gnavus*, *Enterocloster bolteae*, *Lachnoclostridium* sp. *YL 32*, and metabolites, including L-tryptophan, L-glutamic acid, gamma-aminobutyric acid (GABA), and fatty acid esters of hydroxy fatty acids (FAHFAs), as well as disturbed biosynthesis of aromatic amino acids and TCA cycle dysfunction, validated in aged mice. Finally, we employed a support vector machine (SVM) algorithm to construct a machine learning model to classify NA and NCDs groups based on the fusion of EEG data and multi-omics profiles and the model demonstrated 92.69% accuracy in classifying NA and NCDs groups.

**Conclusions:**

Our study highlights the potential of multi-omics profiling and EEG data fusion in personalized diagnosis of NCDs, with the potential to improve diagnostic precision and provide insights into the underlying mechanisms of NCDs.

Video Abstract

**Supplementary Information:**

The online version contains supplementary material available at 10.1186/s40168-023-01717-5.

## Background

Neurocognitive disorders (NCDs) encompass a group of conditions, impairing cognitive functions and affecting quality of daily life [1]. NCDs have a substantial negative societal impact due to their prevalence and associated costs [2]. Approximately 50 million people worldwide suffer from NCDs, but this number is expected to triple by 2050 due to the aging population [3]. Moreover, it is difficult to give personalized diagnosis for NCDs, because the pathology of NCDs is complicated and the symptoms are diverse. Regular diagnostic methods for NCDs, including neuropsychological examination, brain imaging, and laboratory detections, lack of definitive biomarkers or convincing examinations [4]. The development of personalized diagnostic strategies for NCDs, regardless of the overlap in symptoms with other medical and psychiatric conditions, and the heterogeneity in cognitive decline among individuals, is becoming a priority.

Recent multi-omics studies have offered insights into NCDs. Metagenomic studies have revealed alterations in the composition and function of gut microbiota in patients with Alzheimer’s disease (AD), while metabolomics identify altered metabolic pathways, like bile acids and short chain fatty acids (SCFAs) in AD, which are associated with neuroinflammation and cognitive decline [5–7]. Proteomic studies highlight different proteins expression in cerebrospinal fluid, including immune markers, phospholipids, and angiogenic proteins [8]. These findings suggest the potential of multi-omics profiling for investigating NCDs and identifying potential biomarkers [9]. Additionally, multimodal electroencephalography (EEG) can provide information about the timing and sequence of brain activity and has been increasingly employed in the diagnosis of cognitive disorders in recent years, which allows a more comprehensive understanding of brain function and connectivity [10, 11]. In view of these advantages, the fusion of multi-omics profiles and multimodal EEG data has the potential to improve the accuracy of diagnosis for NCDs.

In our previous study, the multi-omics approaches were employed to identify gut microbiota, faecal metabolites, and urine exosomes from seniors with normal cognitive function, as potential predictors of NCDs [12]. However, when the seniors progress to NCDs, their multi-omics profile changes, and the characteristics are still unknown. In this study, to identify specific biomarkers of NCDs, we compared seniors with NCDs to normal aging seniors by rigorous matched cohort and conducted faecal microbiota transplantation (FMT) in aged C57 mice for validation. Meanwhile, we applied machine learning algorithms to integrate multi-omics profile and multimodal EEG data for the development of novel predictive models. It suggests that predictive models can accurately distinguish NCDs patients from the participates contributing to the personalized diagnosis for NCDs.

## Methods

### Ethics statement

The study adheres to the *Declaration of Helsinki* and was approved by the Ethics Committee of the University of Macau (No. BSERE21-APP012-ICMS).

### Study design and subjects

**Fig. 1:**
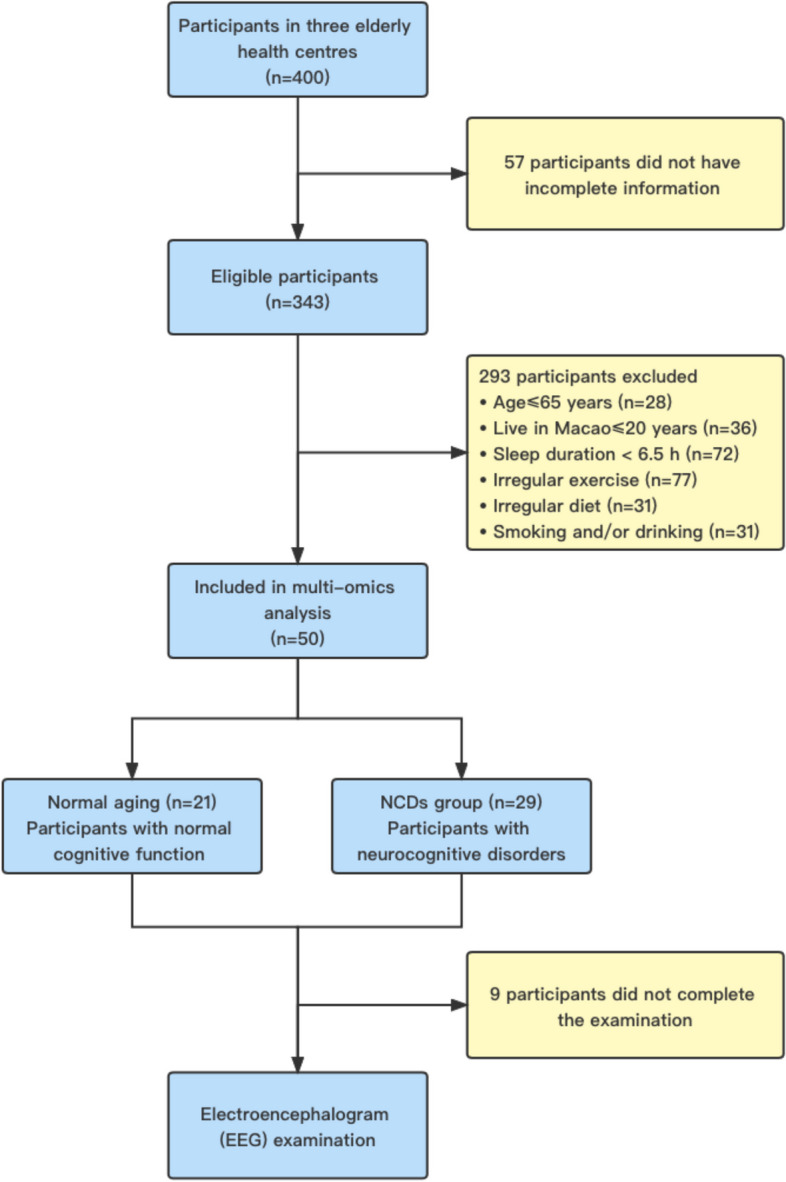
Flowchart of study inclusion/exclusion criteria

Trial recruitment started in September 2019 and 400 seniors were randomly selected from three seniors health centers in Macao Special Administrative Region (Peninsula, Coloane, and Taipa), China. Fifty-seven participants were excluded due to incomplete information. The inclusion criteria for the study were as follows: Chinese residents who have lived in Macao for more than 20 years; no intellectual and language communication barriers, able to understand and answer the questions in Cantonese; and no suffering from major diseases of heart and/or lung in the past year. Exclusion criteria were age less than 65 years old; live in Macao less than 20 years; sleep duration less than six and a half hours, irregular exercise, irregular diet, smoking, and/or drinking; and antibiotics administration within 2 weeks. The study also excluded seniors who suffered from tumor, organ failure, mental illness, and other serious systemic diseases and individuals that failed to complete the questionnaire even with assistance (Fig. 1). The seniors were divided into two groups according to their cognitive function from various domains evaluated by the Hong Kong version of Montreal Cognitive Assessment (MoCA-HK). At the same time, percentile ranking was applied in the interpretation of MoCA-HK scores. If an individual’s MoCA-HK score is lower than 16th percentile compared to their age and education-matched peers, the senior will be classified in the “NCDs group” and vice versa in “normal aging” group [13–15].

### EEG recording and preprocessing

During the EEG recording sessions, all the participants were required to sit comfortably in chair with their eyes opened and were instructed to remain still, avoiding active thought. All participants underwent a twice 5-min session of resting state EEG recording, separated by a 40-min interval. Continuous EEG data were recorded by a 64-channel Biosemi Active Two EEG amplifier system (Biosemi, Amsterdam, Netherlands) with Ag/AgCl scalp electrodes placed based on the international 10–20 system on an elastic cap. Before the EEG recording, the input impedance of all channels was kept below 5 kΩ to ensure the quality. Online EEG recording employed a bandpass filter ranging from 0.01 to 200 Hz, with a sampling rate of 2048 Hz.

The recorded EEG data was preprocessed by MATLAB R2016a (MathWorks, Natick, USA) and EEGLAB. Data were re-referenced to the whole brain and downsampled to 500 Hz. Offline EEG signal processing in EEGLAB included applying a bandpass filter from 1 to 30 Hz. All EEG data were visually inspected to identify and exclude segments with excessive noise, and noisy channels were interpolated using spherical spline interpolation. Additionally, eye movement artifacts (eye blinks and eye movements), muscle artifacts, and heart artifacts were corrected with independent component analysis impeded in EEGLAB.

### EEG power spectral density and EEG microstate analysis

For subsequent EEG power spectral density (PSD) analysis, clean continuous EEG data were segmented into 2-s epochs. Multitaper spectral analysis was used to calculate PSD of each electrode. The absolute power of each channel was then averaged to obtain values for six frequency bands: delta (1–4 Hz), theta (4–8 Hz), alpha1 (8–11.5 Hz), alpha2 (11.5–13 Hz), beta1 (13–21 Hz), beta2 (21–30 Hz). Relative power was obtained by normalizing the power in each frequency band with the overall power in 1–30 Hz range within each channel at the sensor level. This approach allowed us to assess alterations in the frequency distribution of EEG power, capturing a general shift in power from higher to lower frequencies that has been previously reported in individuals with NCDs. Meanwhile, in order to explore the topological distribution of the frequency bands across brain regions, a two-way ANOVA was conducted to investigate hemisphere differences and group differences. Brain regions were categorized as incorporating electrodes (FP1, AF7, AF3, F1, F3, F5, FPZ, FP2, AF8, AF4, AFZ, FZ, F2, F4, F6), central (FC5, FC3, FC1, C1, C3, C5, CP5, CP3, CP1, FC6, FC4, FC2, C2, C4, C6, CP6, CP4, CP2, CPZ), posterior (PO7, PO3, O1, IZ, PZ, POZ, PZ, PO8, PO4, O2), and temporal (F7, FT7, T7, TP7, P1, P3, P5, P7, P9, F8, FT8, T8, TP8, P2, P4, P6, P8) regions in both left and right hemispheres.

EEG microstate analysis was performed using the Microstate toolbox, based on EEGLAB [16]. The cleaned continuous EEG was segmented based on the global field power (GFP) and classified into different classes according to their topography similarity, subsequently. Specifically, to extract GFP peak maps, 1000 GFP peaks were identified into regimentation per participant, with a minimum peak distance of 10 ms. Cluster maps were then calculated based on the newly generated EEG dataset from all participants together. The optimal number of cluster maps was selected from a range of 2 to 8 prototype topography clusters through cross-validation criterion, global explained variance, dispersion, and the Krzanowski-Lai (KL) criterion. For segmentation procedure, the topographic atomize and agglomerate hierarchical clustering (TAAHC) algorithm was chosen as the clustering method, allowing for automatic determination of cluster numbers and polarity. TAAHC started out with all clusters and iteratively removed the “worst” cluster, defined as the one with the lowest sum of correlations to other prototypes. This process continued until a preset minimum number of clusters remained. Once the number of microstate prototypes was selected, the prototypes were backfitted to all the participants using a global map dissimilarity index. To reduce the noise of resting state EEG, unstable EEG topographies within 30 ms were filtered out. After the back-fitting procedure, parameters including microstate duration (average time a microstate remained stable), occurrences (number of times a microstate occurred per second), coverage (proportion of time covered by each microstate), global field power, and transitional probabilities were extracted for further statistical analysis.

### Samples collection from seniors

The faecal and urine sample collection and processing have been described previously [12]. Briefly, the faecal and urine samples were processed within 4h after collection in the laboratory and stored at minus 80°C until analysis. A protease inhibitor cocktail and antibiotics were added to avoid proteolysis and bacterial proliferation in the urine samples. Total DNA was extracted from frozen stools using the QIAamp PowerFecal Pro DNA Kit (QIAGEN, 51,804). Urine exosome isolation was performed through differential ultracentrifugation. The transmission electron microscope (TEM), nanoparticle tracking analysis (NTA), and exosome surface marker proteins ALIX, HSP70, and TSG101 were selected for identification.

### Metagenomic sequencing of intestinal microbiota

The DNA metagenomic shotgun sequencing of the faecal samples was performed as previously described [17]. In brief, for sequencing libraries, VAHTS Universal DNA library preparation kit for Illumina (Vazyme, Nanjing, China) was used, and for qPCR quantification, KAPA SYBR FAST qPCR Kit (Kapa Biosystems, Wilmington, MA, USA) was used. Paired-end on the NovaSeq 6000 instrument (Illumina, San Diego, CA, USA) 2 × 150-bp sequencing was carried out. Following FastQC (v0.12.0) quality control, the sequence reads were pre-processed by HiSAT2 (v2.2.1) and DeconSeq (v0.4.3) to remove human reads and provide clean non-human sequences [18–20]. The ratio of all mapped reads for each species, normalized by all mapped microbial reads and the genome size within each sample, served as a measure of relative abundance. The abundance of microbial BioCyc pathways was examined using the HMP Unified Metabolic Analysis Network (HUMAnN2). Utilizing α diversity and β diversity, respectively, it was determined how different the bacterial communities between the normal aging and NCDs group Chao 1, Simpson and Shannon indexes, and principal coordinate analysis (PCoA) were used to evaluate the diversity. The linear discriminant analysis effective size (LEfSe) was employed to find the significant distinct species.

### Proteomic analysis of urine exosomes

Proteomic analyses were performed using the label-free proteomics method as previously described [21]. In brief, 100 μg of protein were digested for each sample, and the peptide was subsequently desalted using a Phenomenex Strata X C18 SPE column. An Agilent 300 Extend C18 column was used for high pH reverse-phase HPLC to fractionate the sample after that. Peptides were submitted to tandem mass spectrometry (MS/MS) in a Q Exactive™ linked online to the UPLC after being subjected to an NSI source. A total of 2300 V of electrospray voltage were used. At a resolution of 60,000, the orbitrap found whole peptides. Ion fragments were found in the orbitrap at a resolution of 15,000 after peptides were chosen for MS/MS analysis with an NCE setting of 28. The *m/z* scan range for MS scans was 400–1200. The 100 *m/z* fixed initial mass was chosen. Proteins that were differentially expressed were found using fold change ≥ 2 or FC ≤ 0.5 and *P* < 0.05. The SwissProt Human database was searched against tandem mass spectra. The UniPort-GOA database, InterProScan, and GO annotation were employed to classify all detected proteins into cell components, molecular function, and biological process.

### Non-targeted metabolomics of faecal samples

For a non-targeted metabolomics analysis, faecal metabolites were extracted with methanol and analyzed using ultra-high-performance liquid chromatography-tandem mass spectrometry (UHPLC-MS/MS). For each metabolite, peak alignment, peak selecting, and quantitation were carried out using the Compound Discoverer 3.1. According to matching peaks with the mzCloud, mzVault, and Mass List databases, the precise qualitative and relative quantitative results were achieved. Both positive and negative electrospray modes were used to acquire the data. The capillary voltage was 3500 V, and the scan rate was one time per second. The materials’ molecular characteristics were discovered using the Mass Hunter Qualitative Analysis Software (Agilent Technologies) [22]. The metabolites were annotated using the KEGG, HMDB, and LIPID Maps databases. Principal components analysis (PCA) and partial least squares discriminant analysis (PLS-DA) carried out at metaX (v1.2.0) were used to determine the differential metabolites [23]. These metabolites were defined as those with variable importance in the projection (VIP) > 1 and *P* < 0.05 and fold change ≥ 2 or FC ≤ 0.5 [23].

### Faecal microbiota transplantation experiment in mice

Twelve-month-old female C57BL/6J mice were purchased from Vital River Laboratories (Charles River, China) and raised under specific pathogen-free (SPF) conditions. They were acclimatized to a temperature- and humidity-controlled environment (22 ± 2°C, 50 ± 5% humidity) with a 12-h light/dark cycle and given food and water ad libitum for a week before experiments. The mice were randomly divided into three groups (*n* = 10/group): control, NA-FMT (normal aging faecal microbiota transplantation), and NCDs-FMT (neurocognitive disorders faecal microbiota transplantation). To establish the pseudo-germ-free mice model, broad-spectrum antibiotics (ampicillin 1 g/L, neomycin sulfate 1 g/L, and metronidazole 1 g/L) were administered ad libitum in drinking water for 14 consecutive days, with the drinking solution refreshed every 2 days. Faecal microbiota was prepared by diluting 1 g of faeces from seniors with NCDs or normal aging in 10 mL of sterile PBS. Each mouse recipient received 0.2 mL of the suspension via gavage for 10 days after a week of antibiotic solution withdrawal [24]. Following treatment, the mice underwent behavioral experiments, including Y-maze test, novel object recognition test, and Morris water maze test according to previous protocols [25, 26]. After the behavioral experiments, the mice were sacrificed for subsequent metagenomic and metabolomic analyses. Metagenomic analyses was performed as described above and derivatization-liquid chromatography-mass spectrometry was performed for carboxylic acid metabolomics analysis in serum and hippocampus samples [27].

### Machine learning algorithm

The multi-omics data, including gut microbiota, EEG features, metabolomic and clinic data, were utilized to train machine learning models to classify individuals into the normal aging and NCDs group. Initially, all data were normalized to a range between 0 and 1 based on their minimum and maximum values. Given the relatively small sample size and a large number of features, Fisher scores were employed to select one hundred features with highest fisher scores for discriminating the two groups. Fisher score is a univariate feature selection algorithm that is independent of the class distribution and is commonly used to assess the discriminatory power of individual features between two classes of equal probability [28]. After the feature selection stage, the well-established supervised machine learning methods were used to construct the classifier. The supervised machine learning algorithm used in this study was the linear support vector machine (SVM), which determines a linear maximum-margin hyperplane to maximize separation between groups. The machine learning algorithms were implemented in MATLAB (The Math Works, Natwick, MA) and SVM was conducted by LIBSVM (http://www.csie.ntu.edu.tw/~cjlin/libsvm/).

Considering the relatively small sample size, we employed a cross-validation strategy to enhance the model’s generalization ability. One commonly used method in such situations is leave-one-out cross-validation (LOOCV). LOOCV is repeated *N* times (*N* is the number of all subjects, here is 41) and in every iteration one sample is left out to test the classifier and remaining subjects are fed to classifier for training. This procedure is repeated until each subject has been used once as the test sample. Finally, the result for every repetition is averaged to produce the final classification accuracy. To address potential confounding factors related to cross-validation, we also employed tenfold and fivefold cross-validation, which results are presented in the appendix. Four performance metrics including accuracy, sensitivity, specificity, and F1 score were used to assess the performance of the classifier in discriminating normal aging and NCDs group.

In the separate single omics models, the models were constructed as the following specific steps: (1) one hundred features were input into classifier with default SVM settings; (2) then, the top ten highest contributing features were identified within linear SVM model; (3) next, these identified ten features were used for hyperparameter tuning with LOOIC and the hyperparameter tuning were achieved by additional loop flow; (4) based on the results of hyperparameter tunning, we selected the optimal parameters for the linear SVM model with the selected ten features to get the performance of the model, including calculating accuracy, sensitivity, specificity, F1 score, AUC curve and P-R curve. For double omics models, we initially considered 20 features by combining the top ten features from each individual omics analysis. Similarly, for three omics models, we began with 30 features, comprising the ten highest-contributing features from each omics analysis. However, during the model development, including hyperparameter tuning, we consistently worked with a set of ten highest-contributing features to ensure fairness and uniformity across all models, regardless of the number of omics sources involved.

### Statistical analysis

For statistical analysis, the GraphPad Prism 8 and R software 3.7 were utilized. For EEG data, EEG power spectral density was compared by two-way ANOVA to explore two hemisphere difference and group difference. The EEG microstate properties were compared by independent sample *t*-tests. Correlations between six EEG power metrics (delta power, theta power, alpha-1 power, alpha-2 power, beta-1 power, and beta-2 power) and gut microbiota, as well as metabolomic data, were assessed using the Spearman correlation coefficient. Specifically, we included gut microbiota and metabolomic variables which were identified as significantly different in the correlation analysis. Additionally, we explored correlations between gut microbiota diversity at the phylum/genus/species levels and EEG power using Spearman correlation. To account for the potential influence of age, which exhibited significant differences, we controlled for age as a covariate in all comparisons and correlation analyses. The two-sided Wilcoxon’s rank-sum test was employed to compare the diversity and composition of the gut microbiota between the normal aging and NCD group. For all boxplots, the horizontal line represented the median and the box edges the first and third quartiles. The whiskers extended up to 1.5 times the interquartile range. The Kruskal–Wallis (KW) rank sum test and the LEfSe method were used to determine the characteristics of significant differences. LDA > 2.5 and *P* < 0.05 were both accepted as statistically significant values. *P* values, VIP values, and fold change were employed to analyze the expression of different proteins and metabolites. The Benjamini–Hochberg method was applied to manage the false discovery rate (FDR) and the threshold value set to 0.05, used for multiple hypothesis correction for the study of proteomics and metabolomics data. Based on log_2_ (fold change) and − log10 (*P*-value) of the metabolites by ggplot2 in the R language, volcano plots were used to filter the proteins and metabolites of interest. For multiple comparisons in the behavioral experiments, data were analyzed using one-way analysis of variance (ANOVA) followed by the Students-Newman-Keuls (SNK) test. A value of *P* < 0.05 was considered as significant difference.

## Results

### Sociodemographic features and neurocognitive scores of subjects

The study samples consisted of 50 seniors more than 65 years of age screened by living habits, clinical history, no major diseases of heart and/or lung, and no intellectual and communication barriers. Matched samples included 21 subjects in the normal aging group and 29 subjects in NCDs group (Fig. 1). NCDs group can be further divided into three stages, mild NCD, MCI, and major NCD based on their neurocognitive scores. The ratio of sex, age, education level, and neurocognitive scores of the two groups is shown in Table 1. Apart from age and neurocognitive scores, there were no differences between the two groups.

**Table 1:** Sociodemographic features and neurocognitive scores of the study subjects (*n* = 50)

Variable	Normal aging	NCDs group	*P*-value^*^
**Sex – no. (%)**			
Male	3 (14.3)	2 (7.0)	0.638
Female	18 (85.7)	27 (93.0)	
**Age—year**	72.62 ± 3.64	80.31 ± 8.11	0.0002
**Education level – no. (%)**			
Primary schools and below	19 (90.5)	27 (93.0)	> 0.999
Middle school and above	2 (9.5)	2 (7.0)	
**Neurocognitive scores**	23.71 ± 3.64	12.66 ± 4.28	< 0.0001

Notes: Data are expressed as mean ± SD except where frequencies are used for categorical data. ^*^Fisher’s exact test Chi-squared test for categorical variables; *t*-test for continuous variables

### EEG power spectral density and EEG microstate analysis

**Fig. 2:**
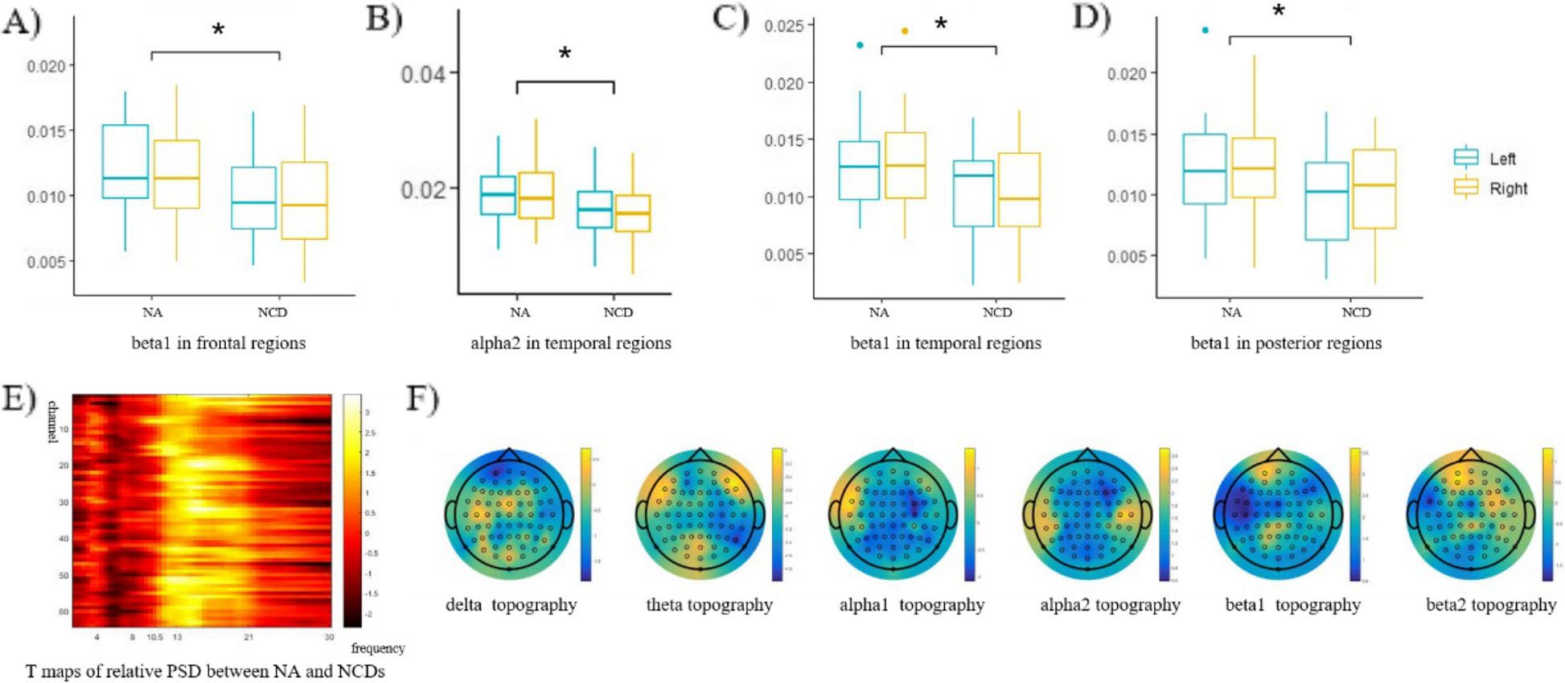
EEG power spectral density and EEG microstate analysis in normal aging (NA) and NCDs group. **A**–**D** The significantly different mean relative PSD of frequency between NA and NCDs group from cortical region of interest (ROI). **E** The relative PSD of frequency across all channels between NA and NCDs groups, column represent the 64 channels and rows represent the frequency bands. **F** The T maps of PSD difference topography in delta, theta, alpha 1, alpha 2, beta 1, beta 2 frequency bands, *P < 0.05, vs. NA group (EEG power spectral density was compared by two-way ANOVA, and EEG microstate properties were compared by independent sample *t*-tests)

The study found significantly decreased relative PSD of alpha and beta frequency bands in NCDs group (Fig. 2). No difference of the relative PSD in left and right hemispheres had been found and no difference of interactions between hemisphere and group had been found, either. In NCDs group, the decreased relative PSD of alpha2 frequency bands in temporal ROI (mean_NCD_ = 0.016, mean_NA_ = 0.021, *Z* = 6.377, *P* = 0.014, p_controlled_ = 0.048) were identified. The beta1 exhibited significantly reduced PSDs in frontal regions (mean_NCD_ = 0.009, mean_NA_ = 0.012, *Z* = 7.065, *P* = 0.009, p_controlled_ = 0.0011), temporal regions (mean_NCD_ = 0.013, mean_NA_ = 0.010, *Z* = 6.958, *P* = 0.010, p_controlled_ = 0.022), and posterior regions (mean_NCD_ = 0.010, mean_NA_ = 0.012, *Z* = 6.009, *P* = 0.0165, p_controlled_ = 0.025). Interestingly, in the two groups, it initially displayed significant differences in the relative PSD of alpha2 (mean_NCD_ = 0.016, mean_NA_ = 0.020, *Z* = 4.851, *P* = 0.030) and beta1 (mean_NCD_ = 0.014, mean_NA_ = 0.012, *Z* = 5.448, *P* = 0.022) frequency bands in the central ROI. However, upon controlling for age as a covariate, the significance of these differences diminished, suggesting that age-related variations may contribute to these EEG patterns. This observation underscores the importance of considering age as a covariate in our analysis to distinguish between age-related changes and those specific to NCDs.

**Fig. 3:**
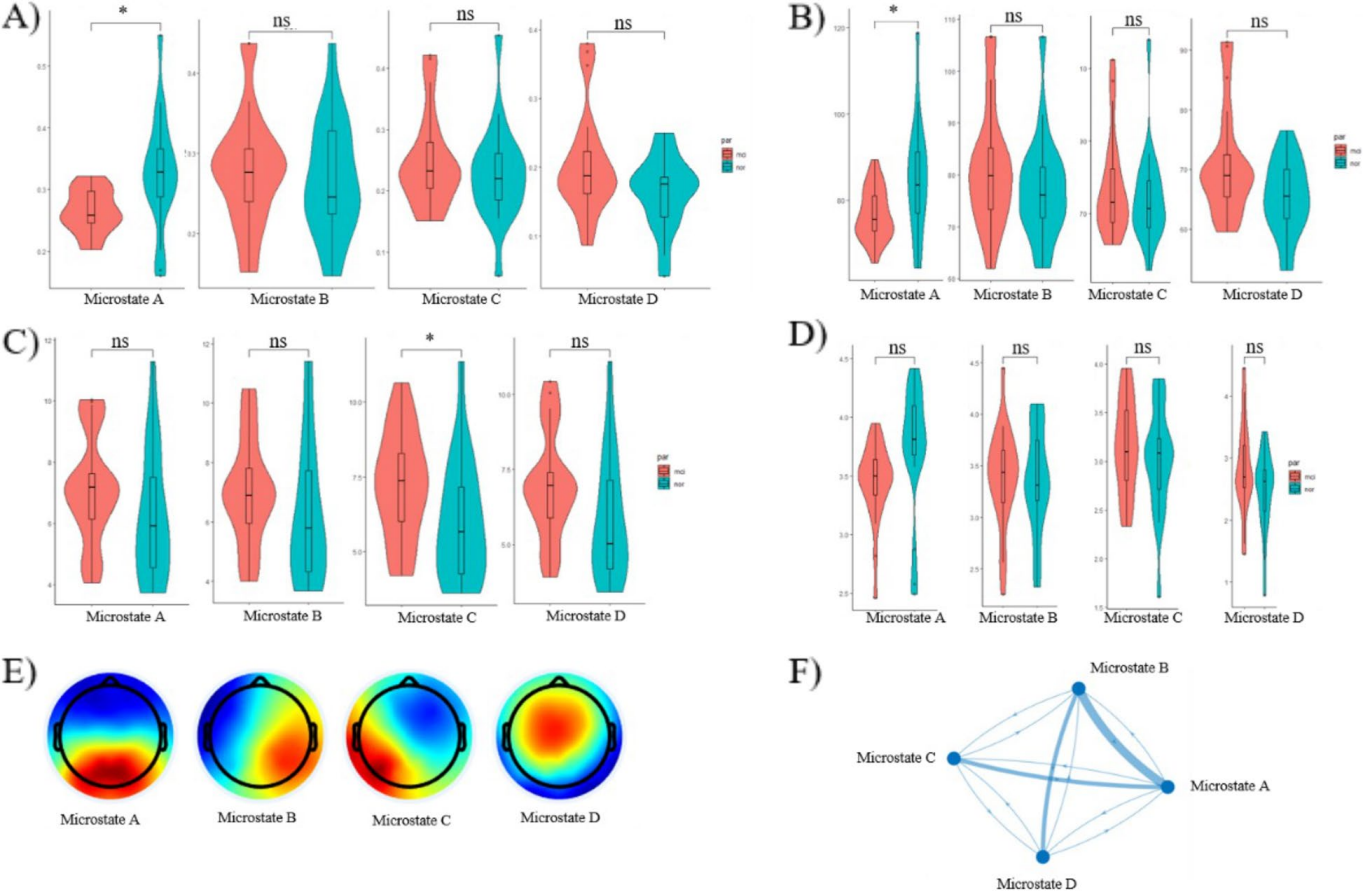
The statistics of microstate A-D property in normal aging and NCDs groups. **A** The coverages of microstate A-D. **B** The durations of microstate A-D. **C** The global field power of microstate A-D. **D** The occurrence of microstate A-D. **E** The prototype topography of microstate A-D. **F** The transitional probabilities among microstates A-D. In Fig. 3F, transitional probabilities among microstates A-D are represented using different line thicknesses. Thin lines indicate no significant difference, medium-thick lines represent a significant difference (*P* < 0.05), and thick lines denote a highly significant difference (*P* < 0.01) in transitional probabilities between normal aging and NCDs groups, as determined by independent sample *t*-tests. *P < 0.05, vs. NA group (independent sample *t*-tests)

In our studies, we have found significantly different microstate properties between normal aging and NCDs groups (Table 2). In NCDs group, it exhibited the decreased coverage property in microstate A (mean_NCD_ = 0.264, mean_NA_ = 0.326, *T* =  − 2.724, *P* = 0.012, p_controlled_ = 0.033) when compared with normal aging group (Fig. 3A). Similarly, compared with normal aging group, in NCDs group, it presented the reduced microstate durations in microstate A (mean_NCD_ = 76.749, mean_NA_ = 85.054, *T* =  − 2.533, *P* = 0.017, p_controlled_ = 0.045, Fig. 3B). While for GFP property, only microstate C was found significant different with increased GFP in NCDs group (mean_NCD_ = 7.234, mean_NA_ = 5.988, *T* = 2.046, *P* = 0.048, p_controlled_ = 0.023, Fig. 3C). Meanwhile, when age is controlled as a covariate, no difference has been found in occurrence or in altered microstate D property coverage (mean_NCD_ = 0.206, mean_NA_ = 0.164, *T* = 2.041, *P* = 0.040, p_controlled_ = 0.13, Fig. 3A) or duration (mean_NCD_ = 70.846, mean_NA_ = 65.219, *T* = 2.35, *P* = 0.024, p_controlled_ = 0.13, Fig. 3D), either. EEG microstates exhibited stable configurations for brief periods before transitioning into other microstates, then we can get their transitional probabilities. For the transitional probabilities (Fig. 3, F), in the NCDs group, we observed a lower probability of transitioning from microstate B (mean_NCD_ = 0.3715, mean_NA_ = 0.4549, *T* =  − 3.292, *P* = 0.002, p_controlled_ = 0.148). However, when controlling for age as a covariate, no significant difference in transitional probabilities was found for microstate C to microstate A (mean_NCD_ = 0.366, mean_NA_ = 0.4234, *T* =  − 2.568, *P* = 0.014, p_controlled_ = 0.07). Additionally, we noted a higher probability of transitioning from microstate B to microstate D (mean_NCD_ = 0.287, mean_NA_ = 0.234, *T* = 2.211, *P* = 0.033, p_controlled_ = 0.078).

**Table 2:** The statistics of microstate A-D property in normal aging and NCDs groups

		**Microstate A**				**Microstate B**				**Microstate C**				**Microstate D**			
		Mean (SD)	T	p1	p2	Mean (SD)	T	p1	p2	Mean (SD)	T	p1	p2	Mean (SD)	T	p1	p2
**Coverage**	NA	0.326 (0.093)	2.724	0.012*	0.033*	0.267 (0.073)	−0.62	0.54	0.90	0.228 (0.080)	−0.73	0.411	0.459	0.164 (0.053)	−2.04	0.04*	0.13
	NCDs	0.264 (0.035)				0.281 (0.073)				0.248 (0.077)				0.206 (0.077)			
**Duration**	NA	85.054 (13.048)	2.553	0.017*	0.045*	77.375 (10.305)	−1.14	0.26	0.787	74.416 (13.196)	−0.72	0.475	0.62	65.219 (6.337)	−2.35	0.024*	0.13
	NCDs	76.749 (5.966)				81.293 (11.658)				77.474 (13.889)				70.846 (8.928)			
**GFP**	NA	6.178 (2.123)	−1.53	0.136	0.473	6.199 (2.333)	−1.27	0.21	0.567	5.988 (2.053)	−2.05	0.048*	0.023*	5.814 (2.076)	−1.86	0.07	0.35
	NCDs	7.118 (1.775)				0.732 (1.800)				7.234 (1.808)				6.955 (1.796)			
**Occurrence**	NA	3.741 (0.546)	2.065	0.048*	0.134	3.355 (0.522)	−0.19	0.86	0.806	3.011 (0.548)	−0.87	0.390	0.287	2.464 (0.646)	−1.73	0.091	0.132
	NCDs	3.442 (0.338)				3.384 (0.482)				3.152 (0.475)				2.835 (0.723)

### Differential gut microbiota compositions and pathway prediction

**Fig. 4:**
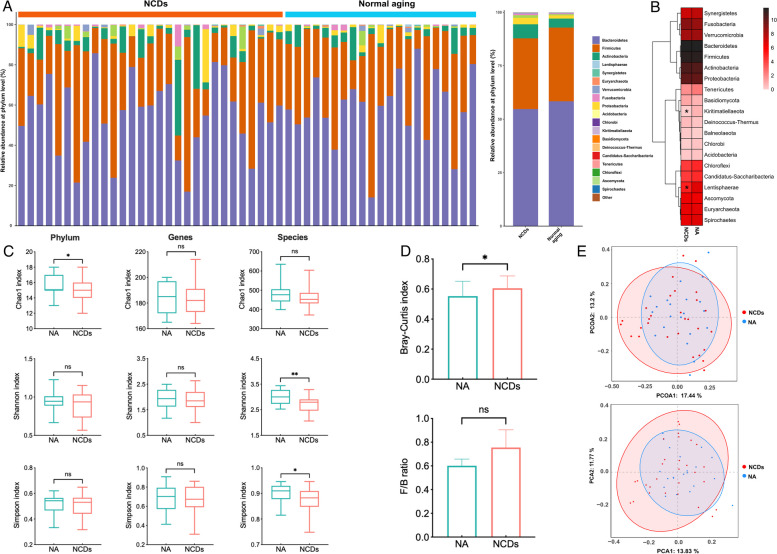
Gut microbiota analysis in normal aging and NCDs groups. **A** Stacked bar chart showed the relative and average relative abundance of gut microbiota at the phylum level between the two groups. **B** Heatmap analysis on the relative abundance of gut microbiota at the phylum level. **C** Alpha diversity analysis, as measured by Chao1 index, Shannon index, and Simpson index, at the phylum, genus, and species levels. **D** Beta diversity analysis, measured by Bray–Curtis dissimilarity, with *P* values computed using a two-sided Wilcoxon test. Additionally, the *Firmicutes/Bacteroidetes* (F/B) ratio between the normal aging and NCDs groups was presented. **E** Principal coordinate analysis (PCoA) and principal component analysis (PCA) of the gut microbiota at the species level showed the separation between the two groups. *P < 0.05, vs. NA group (two-sided Wilcoxon’s rank-sum test)

At the phylum taxonomic rank, the predominant taxa detected in the gut microbiota of both cohorts were *Bacteroidetes*, *Firmicutes*, *Actinobacteria*, and *Proteobacteria*, collectively representing up to 98% of the overall relative abundance (Fig. 4A). The heatmap of the top 20 phyla, based on their relative abundances, revealed a significant reduction in the prevalence of *Kiritimatiellaeota* and *Lentisphaerae* in the NCDs group when compared to the normal aging cohort (Fig. 4B). The assessment of α diversity was conducted using Chao1, Shannon, and Simpson indices to evaluate the richness and diversity of the microbiota, as presented in Fig. 4C. The analysis revealed a significant decrease in diversity indices at the phylum and species levels among the NCDs group, with statistically significant differences observed in the Chao1 index at the phylum level, as well as the Shannon and Simpson indices at the species level. At the genus level, we observed a notable trend towards a decrease in alpha-diversity. The assessment of β diversity was performed using Bray–Curtis dissimilarities to evaluate the differences in diversity between two groups. The analysis revealed a statistically significant increase in Bray–Curtis dissimilarities within the group with normal aging, as compared to the group with NCDs. In addition, this change became more pronounced as the degree of NCDs progressed (Supplementary Fig. S1). Despite observing an increase in the *Firmicutes/Bacteroidetes* (F/B) ratio in NCDs group as compared to the group with normal aging, this change did not reach statistical significance (Fig. 4D). The results of the principal component analysis (PCA) and principal coordinates analysis (PCoA) of species composition indicated an observable clustering and discernible trends between the group with normal aging and the group with NCDs, implying that the dysbiosis of gut microbiome was linked with NCDs (Fig. 4E).

**Fig. 5:**
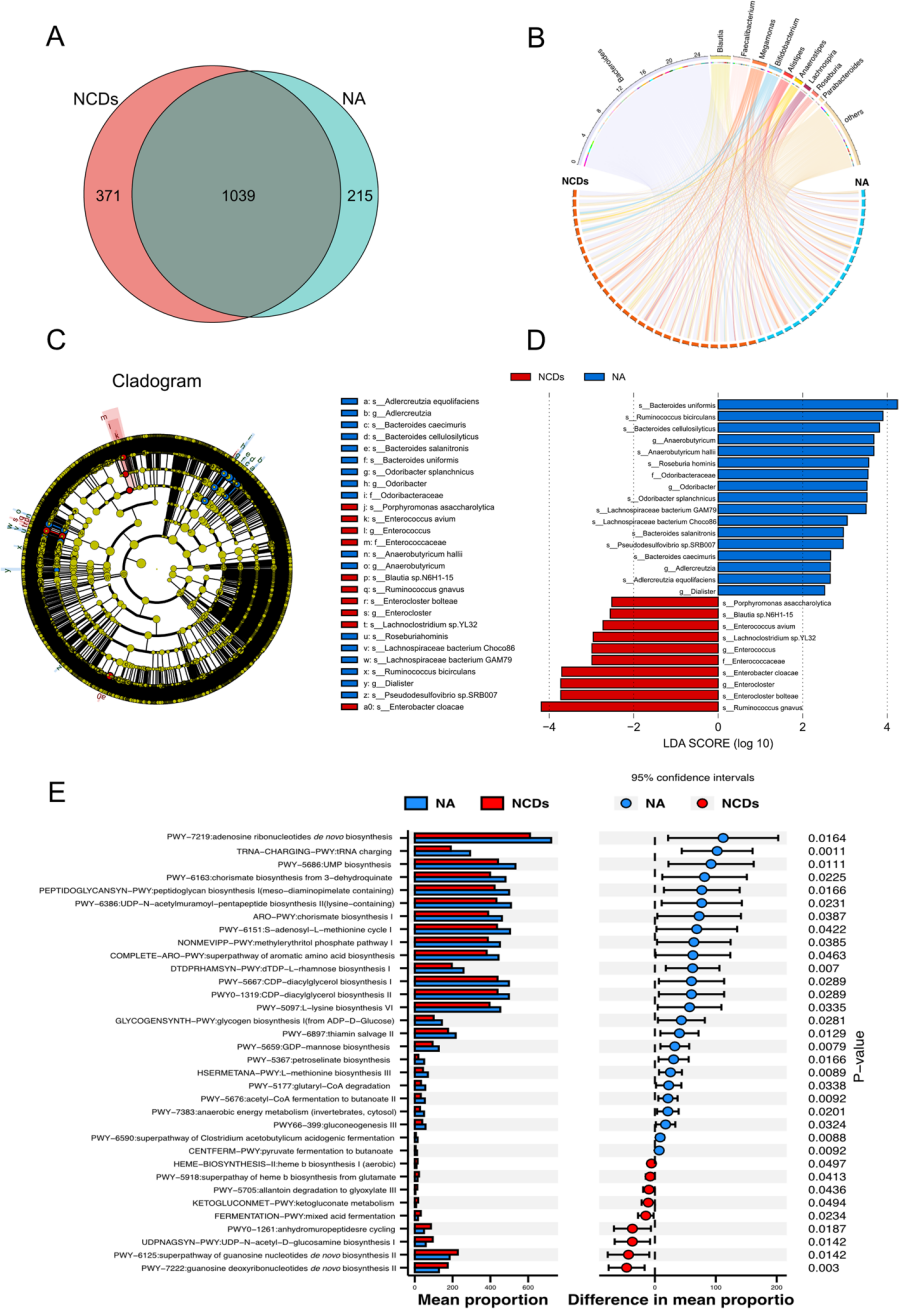
Significant shifts in gut microbial compositions at the species level and related predictive bacterial functional profiles. **A** Venn diagram illustrated the overlap of gut microbiota species between the normal aging and NCDs groups. **B** The chord diagram showed the abundance of gut microbiota at the genus level and their relationships between the normal aging and NCDs groups. **C** Cladogram representation of the linear discriminant analysis (LDA) effect size (LEfSe) analyses of gut microbiota, with an LDA score > 2.5. **D** Distinctive gut microbiota composition associated with the NCDs group was revealed by the LEfSe analysis. **E** Comparative analysis of the relative abundances of BioCyc pathways between the normal aging and NCDs groups. The STAMP analysis was applied to identify significant differential abundant BioCyc pathways. The Kruskal–Wallis (KW) rank sum test and the LEfSe method were used to determine the characteristics of significant differences

Furthermore, the Venn diagram was utilized to illustrate the unique and share the species of gut microbiota for a better understanding of their richness. The analysis showed that a total of 1039 operational taxonomic units (OTUs) were common to all samples, while 215 OTUs and 371 OTUs were specific to normal aging and NCDs groups, respectively (Fig. 5A). To depict the connections and relative abundances of the top ten enriched gut microbiota species between the two groups, a Chord diagram was generated. The arcs in the diagram represented connections that were proportional to the size of each arc, while node segments along a circle represented the genus and the node size reflected the abundance of the contributing genus (Fig. 5B). LEfSe analysis was performed to identify differential microbiota species between normal aging and NCDs groups. A cladogram obtained from the LEfSe analysis showed the phylogenetic distribution of the microbiota (Fig. 5C). The analysis revealed 17 bacterial taxa enriched in normal aging and 10 bacterial taxa enriched in the NCDs group. Additionally, LEfSe analysis identified 7 species-level bacterial signatures specific for NCDs, including *Ruminococcus gnavus*, *Enterocloster bolteae*, *Enterobacter cloacae*, *Enterococcus avium*, *Porphyromonas asaccharolytica*, *Blautia* sp. *N6H1-15*, and *Lachnoclostridium* sp. *YL 32* (Fig. 5D). In this study, PICRUSt was utilized to predict the functional content of the metagenome by leveraging metagenomic shotgun sequencing and BioCyc pathway analysis. The BioCyc pathways represent a collection of coordinated biological processes constructed based on the information present in the BioCyc database. The results of STAMP analysis indicated that the abundance of pathways related to the biosynthesis of aromatic amino acids, namely PWY-6163, ARO-PWY, and COMPLETE-ARO-PWY, as well as those associated with the tricarboxylic acid cycle (TCA cycle), specifically PWY-7219, PWY-6897, PWY-7383, and PWY66-399, exhibited a significant decrease in the NCD group when compared to the normal aging (Fig. 5E).

### Differential urine exosomes proteins

**Fig. 6:**
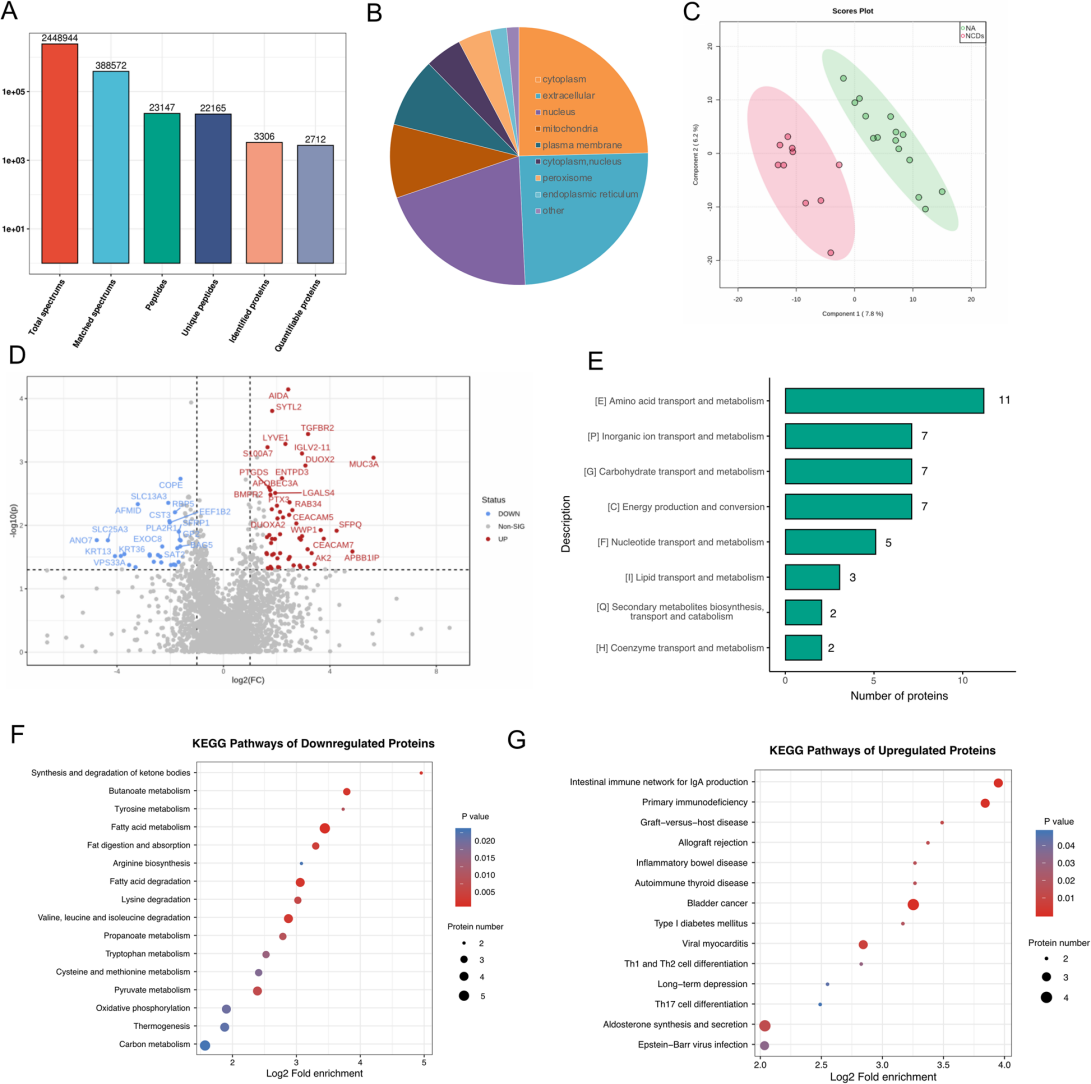
Quantitative proteomics analysis on urine exosomes using label-free method. **A** Overview of protein identification and the number of identified spectra, peptides, and proteins after data filtering for search library results. **B** Gene Ontology (GO) annotation of the subcellular locations of the identified proteins. **C** Scores plot obtained from partial least squares discriminant analysis (PLS-DA) of identified proteins. **D** Volcano plot of differentially expressed proteins between the normal aging and NCDs groups, where red indicated upregulated proteins and blue indicated downregulated proteins. **E** Clusters of Orthologous Groups (COG)/eukaryotic orthologous groups (KOG) analysis of metabolic functions of differentially expressed proteins. Comparing the bubble plot of the significantly enriched KEGG pathways of downregulated proteins (**F**) and upregulated proteins (**G**) between the normal aging and NCDs groups. Proteins that were differentially expressed were found using fold change ≥ 2 or FC ≤ 0.5. The Benjamini–Hochberg method was applied to manage the false discovery rate (FDR) and the threshold value set to 0.05. Proteins with a corrected *P*-value < 0.05 were considered significant

To investigate the differences in protein expression between normal aging and NCDs groups, and to validate metagenomics predicted pathways, we utilized urine samples, a commonly used non-invasive examination method, to isolate exosomes through ultracentrifugation and further purified them (Supplementary Fig. S2). Proteins were then extracted and analyzed through mass spectrometry resulting in 2,448,944 secondary spectrograms, 388,572 of which were analyzed to identify 23,147 peptide segments with 22,165 specific segments. Of these segments, 3306 proteins were identified, with 2712 quantifiable (Fig. 6A). These proteins were localized in various subcellular regions such as the cytoplasm, extracellular space, nucleus, and mitochondria as determined by Gene Ontology (GO) annotation (Fig. 6B). PLS-DA analysis indicated the differences in protein distribution between the NCDs and normal aging groups (Fig. 6C). Differential abundance of proteins was evaluated using a fold-change criterion of > 1.5 (upregulated) or < 1.5 (downregulated) and a *P*-value of < 0.05. The volcano map suggested that red represented significantly upregulated differential proteins and blue indicated significantly downregulated differential proteins in the NCD group compared to the control group, with 57 and 30 proteins, respectively (Fig. 6D). Metabolic function analysis through Clusters of Orthologous Groups (COG)/eukaryotic orthologous groups (KOG) revealed that differentially expressed proteins were predominantly involved in amino acid transport and metabolism, and energy production and conversion, in agreement with the predicted results of the metagenomics analysis (Fig. 6E). Furthermore, KEGG pathway enrichment analysis confirmed these findings. Pathways enrichment analysis of downregulated urine exosome proteins showed that tyrosine metabolism, tryptophan metabolism, and pyruvate metabolism were reduced in the NCDs group (Fig. 6F). Conversely, pathways associated with diseases such as inflammatory bowel disease, bladder cancer, type I diabetes mellitus, and long-term depression were enriched in the upregulated proteins of the NCDs group (Fig. 6G).

### Differential faecal metabolites

**Fig. 7:**
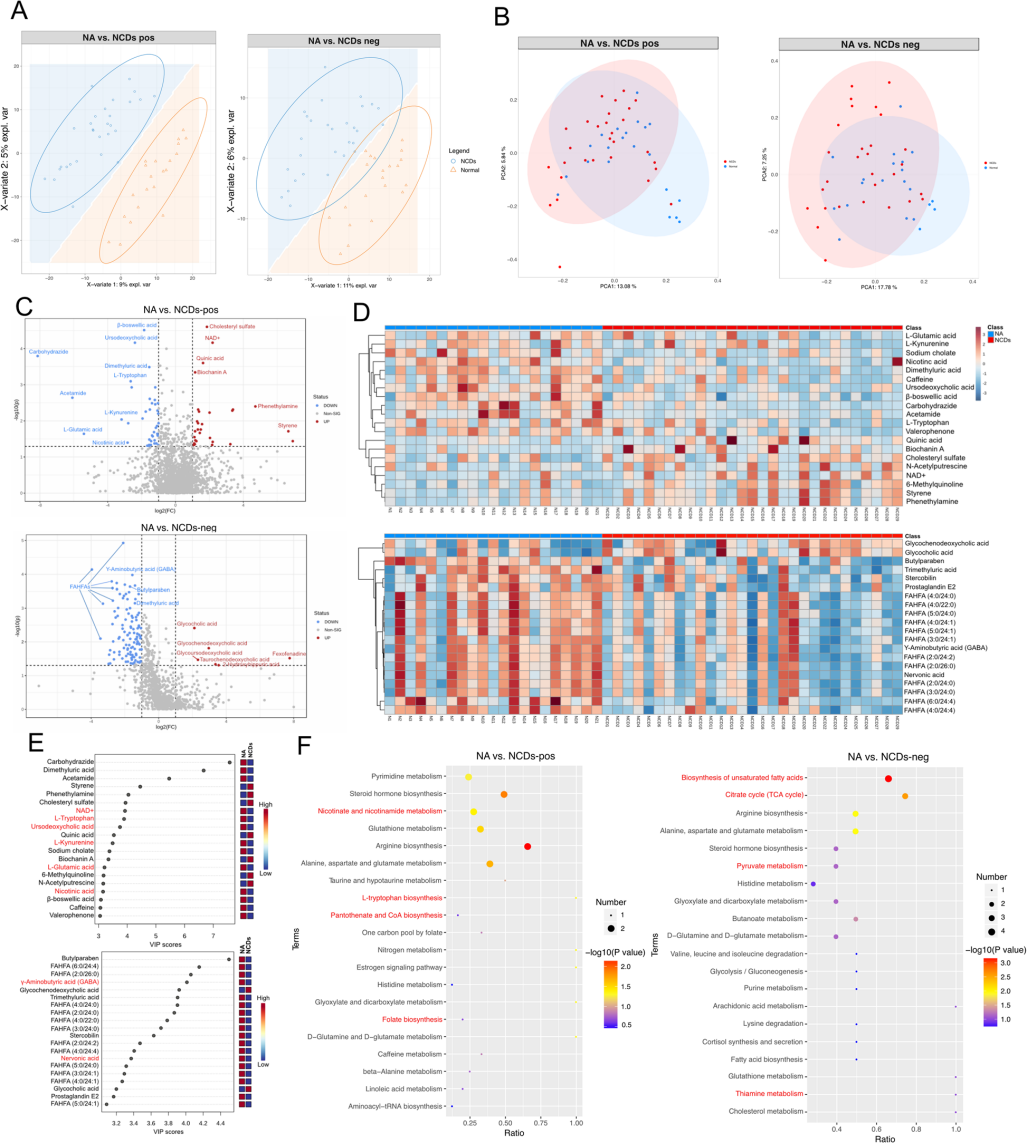
Metabolomic profiling of faecal samples in normal aging and NCDs groups. **A** PLS-DA score plot of primary metabolites detected in positive and negative ion modes, indicating a clear separation between the two groups. **B** PCA of the primary metabolites detected in positive and negative ion modes, confirming the discrimination between the two groups. **C** Volcano plot of differentially expressed metabolites in positive and negative ion modes between the normal aging and NCDs groups, with blue dots representing downregulated metabolites and red dots representing upregulated metabolites. **D** Heatmap of significantly differentially expressed metabolites based on adjusted *P*-values and fold changes. **E** Variable importance plot from random forest model of significantly differentially expressed metabolites based on VIP values, showing the most influential metabolites for group classification. **F** Bubble plot of the top 20 significantly enriched KEGG pathways between the normal aging and NCDs groups in negative and positive ion modes, revealing the metabolic pathways that were most affected by NCDs. The metabolites with variable importance in the projection (VIP) > 1 and *P* < 0.05 and fold change ≥ 2 or FC ≤ 0.5 were differential metabolites. Volcano plots were used to filter metabolites of interest which based on log_2_(FoldChange) and − log_10_(*p*-value) of metabolites by ggplot2 in R language. When *P*-value of metabolic pathway < 0.05, metabolic pathway was considered as statistically significant enrichment

After validation of the proteomics data, the subsequent step involves conducting metabolomics analysis to further corroborate and elucidate the observed differences in protein expression between the normal aging and NCDs groups, as well as to gain a more comprehensive understanding of the underlying molecular mechanisms involved in age-related diseases. A PLS-DA score plot of primary metabolites detected in positive and negative ion modes indicated a clear separation between the normal aging and NCDs groups (Fig. 7A). PCA of the primary metabolites detected in positive and negative ion modes confirmed the discrimination between the two groups (Fig. 7B). A total of 54 and 54 metabolites were significantly upregulated and downregulated, respectively, in the positive ion model compared with the normal aging, while 12 and 63 metabolites were significantly upregulated and downregulated, respectively, in the negative ion model (Fig. 7C). The top 20 upregulated and downregulated differential metabolites were screened, and the hierarchical clustering analysis classified the metabolites based on their characteristics between the two groups, as visualized in a heatmap (Fig. 7D). The top 20 metabolites were further delineated based on VIP values, and variable importance plots were constructed. The results showed that NAD + , L-tryptophan, ursodeoxycholic acid, L-kynurenine, L-glutamic acid, nicotinic acid, γ-aminobutyric acid (GABA), and nervonic acid were downregulated in the NCDs group. A significant decrease in fatty acid esters of hydroxy fatty acids (FAHFAs) in faecal metabolites were also observed in seniors with NCDs (Fig. 7E). KEGG annotation analysis was conducted to identify all pathways of differential metabolites, and the results showed that nicotinate and nicotinamide metabolism, L-tryptophan biosynthesis, pantothenate and CoA biosynthesis, folate biosynthesis, biosynthesis of unsaturated fatty acids, citrate cycle, pyruvate metabolism, and thiamine metabolism were associated with NCDs (Fig. 7F).

### Experimental verification in FMT mice

**Fig. 8:**
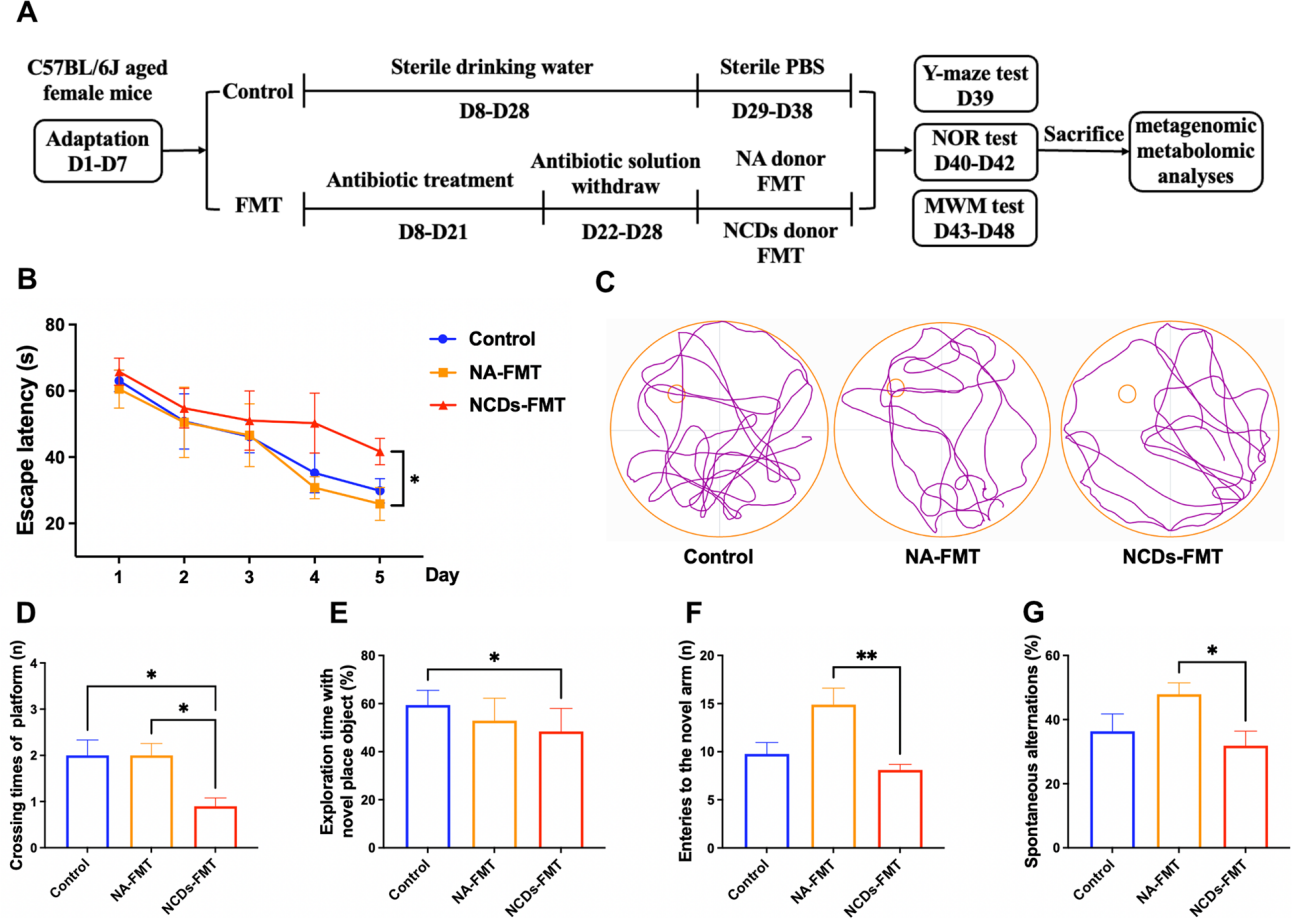
Evaluation of spatial learning and memory performance in FMT mice using the Y-maze test, novel object recognition test, and Morris water maze test (*n* = 8–10). **A** The diagram for the experimental design. **B** The escape latency time to reach the hidden platform during training days. **C** The representative track plots of 3 groups. **D** The number of entries in the platform zone during the probe trial in the MWM test. **D** The percentage of time spent with the object in the novel place to the total object exploration time in NOR test. **F** The number of novel arm entries and (**G**) percentage of spontaneous alternations in the Y-maze test. Data are presented as means ± SEM (*n* = 8–10). *P < 0.05, **P < 0.01 vs. the NCDs-FMT group (one-way ANOVA followed by Student–Newman–Keuls test)

To investigate the potential impact of gut microbiota alterations on cognitive function, we conducted a FMT experiment in a pseudo-germ-free mouse model, followed by behavioral tests including the Y-maze test, novel object recognition (NOR) test, and Morris water maze (MWM) test. To further validate the metabolic pathways, targeted metabolomics analyses of hippocampal and serum samples were performed (Fig. 8A). In the MWM test, mice were trained to locate a hidden platform, and the escape latency to reach the platform gradually decreased during the training process. Compared to the normal aging (NA)-FMT mice, NCDs-FMT mice exhibited a significantly longer escape latency on day 5 (*P* < 0.05) and impaired memory in the probe trial, as evidenced by a significant decrease in the number of times crossing the target quadrant (*P* < 0.05, Fig. 8B–D). In the NOR test, NCDs-FMT mice spent significantly less time exploring the novel object compared to the total object exploration time than the control group (*P* < 0.05, Fig. 8E). The Y-maze test results suggested that NCDs-FMT mice were less likely than NA-FMT mice to explore novel and alternate arms, reflecting impaired immediate spatial working memory performance (*P* < 0.01 and *P* < 0.05, respectively, Fig. 8F, G). Taken together, the behavioral experimental results suggest that FMT of seniors with NCDs impaired spatial learning and memory in aged mice.

**Fig. 9:**
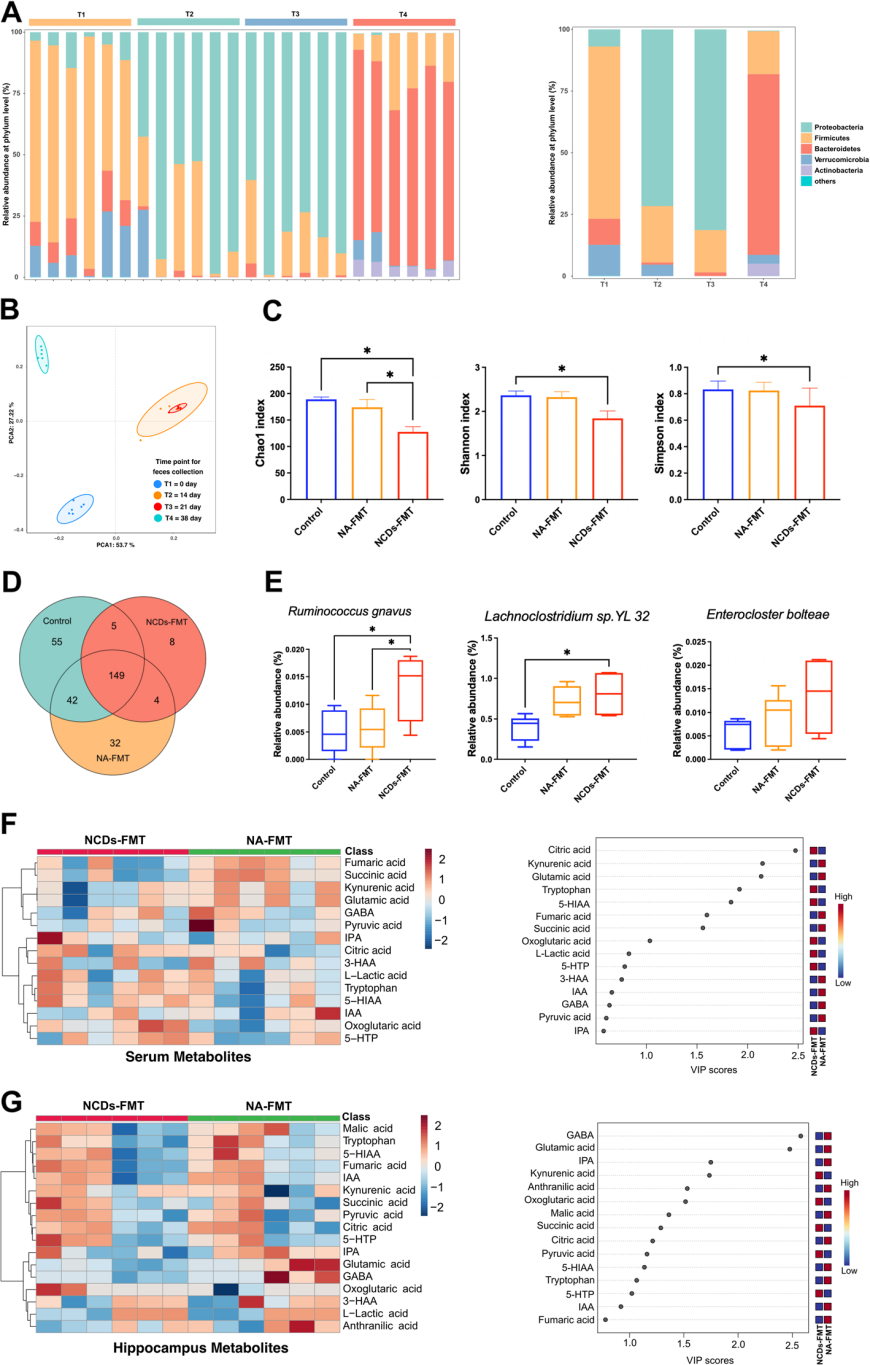
Evaluation of metagenomic analysis of gut microbiota and metabolomic analysis of serum and hippocampus in faecal microbiota transplantation (FMT) mice. **A** Stacked bar chart illustrated the relative and average relative abundance of gut microbiota at the phylum level at different time points: days 0, 14, 21, and 38 (T1, T2, T3, and T4). **B** Principal component analysis (PCA) of gut microbiota composition suggested the distances at T1, T2, T3, and T4. **C** Alpha diversity analysis of gut microbiota at the species level, as determined by Chao1 index, Shannon index, and Simpson index. **D** Venn diagram showed the gut microbiota species present. E The changes in relative abundance of signature gut microbiota species of seniors with NCDs in FMT mice. F Heat maps and variable importance plots of serum metabolites. G Heat maps and variable importance plots of hippocampus metabolites. The row dendrograms were generated using hierarchical clustering with the Euclidean distance metric

In this study, the impact of FMT on the gut microbiota was evaluated through the collection and analysis of faecal pellets at different time points. Prior to FMT, baseline faecal samples were collected (T1), followed by antibiotic administration at days 14, 21, and 38 (T2, T3, and T4). The abundance of two major bacterial phyla, Firmicutes and Bacteroidetes, decreased significantly within 2 weeks of antibiotic treatment, indicating a substantial depletion of the gut microbiota (Fig. 9A). The gut microbiota composition was analyzed through principal component analysis (PCA), which showed a clear separation between samples obtained before and after antibiotic intervention (Fig. 9B). Furthermore, the diversity of the bacterial community at the species level was assessed using the Chao1, Shannon, and Simpson indices. The analysis revealed a significant decrease in the α-diversity index in NCDs-FMT mice (*P* < 0.05, Fig. 9C). A Venn diagram showed that a total of 149 operational taxonomic units (OTUs) were common to all samples, while 32 OTUs and 8 OTUs were specific to NA-FMT mice and NCDs-FMT mice, respectively (Fig. 9D). The identification of bacterial biomarkers for NCDs revealed that *Ruminococcus gnavus*, *Enterocloster bolteae*, and *Lachnoclostridium* sp. *YL 32* were three of the seven species-level bacterial biomarkers identified by LEfSe analysis. The relative abundance of *Ruminococcus gnavus* and *Lachnoclostridium* sp. *YL 32* increased significantly in mice, consistent with the population samples. The metabolic pathways initially predicted by metagenome sequencing, including the biosynthesis of aromatic amino acids and the TCA cycle, were validated against the results of serum and hippocampal metabolomics of mice. The relative content of citric acid, fumaric acid, succinic acid, oxoglutaric acid, pyruvic acid, and tryptophan all changed significantly (Fig. 9F, G). Notably, the relative content of citric acid was significantly increased in serum but significantly decreased in the hippocampus.

### Machine learning classification

**Fig. 10:**
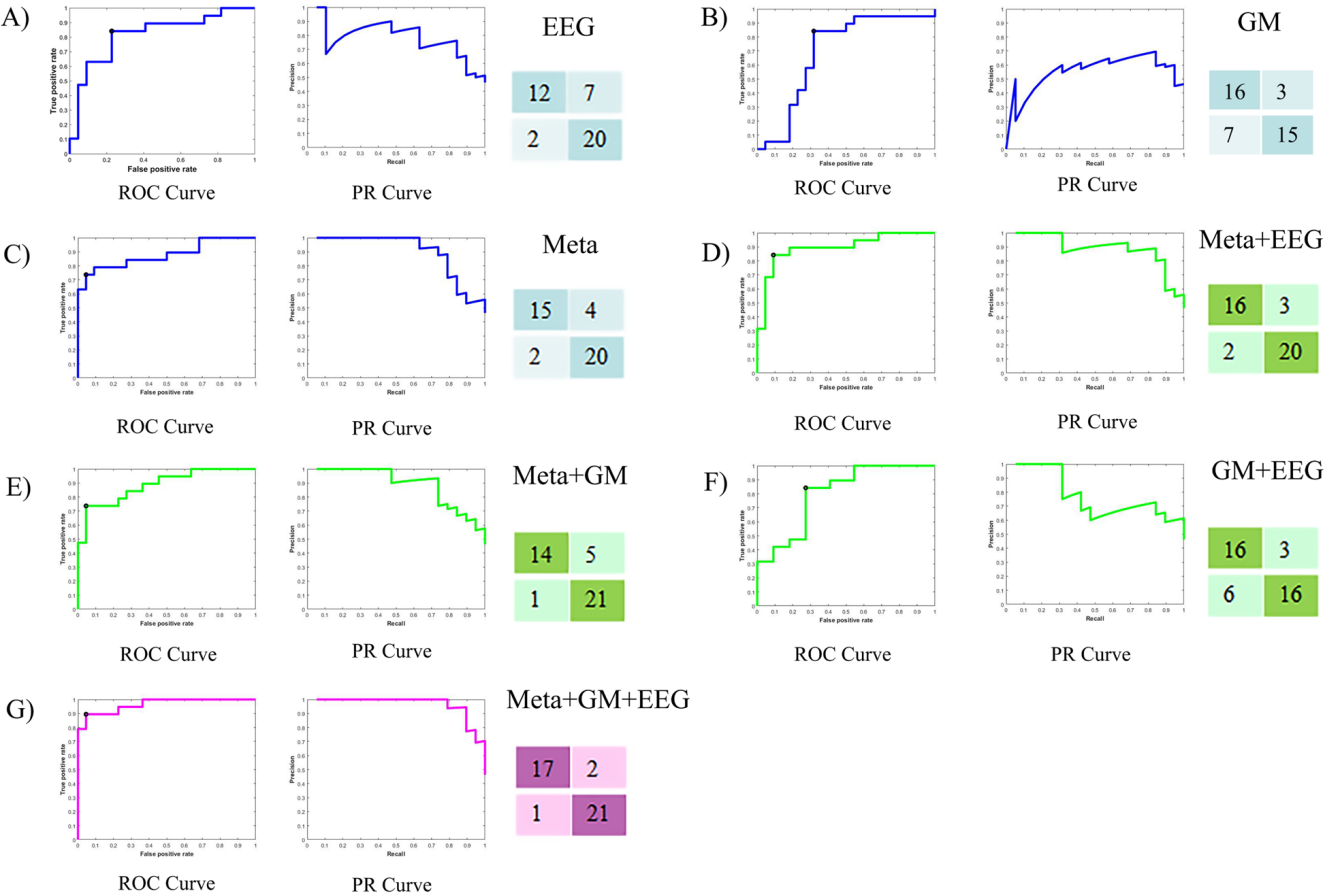
Machine learning performance of different omics. In each panel, the left is the ROC curve, and middle is P-R curve, while the matrix represented the difference between predicted label and true label. **A**–**C** The single omics discriminated the NA and NCDs group in blue. **D**–**F** The double omics discriminated the NA and NCDs group in green. **G** The classification of multi-omics. *Meta* Metabolomic, *GM* Gut microbiota

The SVM was used to construct the machine learning model to classify the normal aging and NCDs groups, based on the feature gained from three omics-EEG, metabolomic and gut microbiota (Table 3 and Fig. 10). In single omics, the SVM achieved the accuracy of 78.05%, 85.71% of precise rate, 63.16% recall rate, and 0.8182 AUC value based on EEG (Fig. 10A), reaching the accuracy of 75.61%, 66.67% of precise rate, 89.47% recall rate, and 0.7895 AUC value based on gut microbiota (Fig. 10B), obtaining the accuracy of 82.96%, 87.50% of precise rate, 73.68% recall rate, and 0.8780 AUC value based on metabolomic (Fig. 10C). While in two omics, the SVM achieved the accuracy of 87.81%, 88.89% of precise rate, 84.21% recall rate, and 0.8947 AUC value based on EEG combining metabolomic (Fig. 10D), reaching the accuracy of 78.05%, 72.73% of precise rate, 84.21% recall rate, and 0.8014 AUC value based on EEG combining gut microbiota (Fig. 10E), obtaining the accuracy of 85.37%, 93.33% of precise rate, 73.68% recall rate, and 0.8852 AUC value based on gut microbiota combining metabolomic (Fig. 10F). Eventually, the multi-omics approach reached an accuracy of 92.69%, 94.44% of precise rate, 89.47% recall rate, and 0.9641 AUC (Fig. 10G). With the source of omics increasing, the performance of the model rose gradually using the same number of features.

**Table 3:** The machine learning performance of different omics

Modality	ACC	Precise	Recall	F1 score	AUC	PR-AUC	Optimal operating point
EEG	78.05%	85.71%	63.16%	0.7273	0.8182	0.7328	(0.2273, 0.7368)
Gut microbiota	75.61%	66.67%	89.47%	0.7826	0.7895	0.6577	(0. 4091, 0.9474)
Metabolomic	82.93%	87.50%	73.68%	0.8000	0.8780	0.8490	(0.0455, 0.7368)
EEG + metabolomic	87.81%	88.89%	84.21%	0.8649	0.8947	0.8364	(0.0909, 0.8421)
EEG + gut microbiota	78.05%	72.73%	84.21%	0.7805	0.8014	0.7252	(0.1346, 0.6842)
Metabolomic + gut microbiota	85.37%	93.33%	73.68%	0.8235	0.8852	0.8388	(0.0455, 0.7368)
EEG + metabolomic + gut microbiota	92.69%	94.44%	89.47%	0.9198	0.9641	0.9136	(0.0455, 0.9474)

## Discussion

Due to the worldwide aging population, the prevalence of NCDs is rising. Diagnosis of NCDs requires the medical history, symptoms, neuropsychological testing, and appropriate objective assessments, using standard criteria such as DSM-5 depending on the type of NCDs [29]. However, the traditional diagnostic approaches for NCDs, such as clinical and neuropsychological assessments, have limitations in terms of accuracy and reliability [30, 31]. Furthermore, previous studies have proposed artificial intelligence model via utilizing biological or neuroimaging biomarkers [32, 33]. However, individuals diagnosed with NCDs usually show a high level of heterogeneity in clinical trajectory, symptoms, as well as neurodegenerative biomarkers [34]. Such heterogeneity is highly associated with the etiology of disease, and the previous models were constructed by single omics, which fails to address the heterogeneity problems [33]. Therefore, the development of novel approaches that utilize multi-omics data, such as gut microbiota, metabolomics, and EEG, can provide valuable insights into the pathophysiological characteristics of NCDs and assist in developing accurate models for personalized diagnosis.

The present study found that seniors with NCDs exhibited significantly decreased relative PSD in the alpha and beta frequency bands during resting state EEG, as well as altered microstate properties compared to healthy controls. These findings are consistent with previous research demonstrating that individuals with cognitive impairment or dementia exhibit lower alpha and beta power compared to healthy controls [35, 36]. Specifically, reductions in alpha and beta power have been linked to deficits in attention, memory, executive function, and rhythmic behaviors [37, 38], while alterations in microstate properties have been associated with disrupted neural synchronization and impaired cognitive processing [39, 40]. The present results suggest that the observed changes in EEG activity may reflect underlying neural dysfunction in individuals with NCDs. It is possible that the decreased relative power in the alpha and beta frequency bands reflects reduced neuronal activity or impaired neural communication in these regions, which could contribute to cognitive deficits observed in this population [41]. Similarly, alterations in microstate properties may reflect disruptions in the neural networks that support cognitive processing, which could further exacerbate cognitive impairment [42]. These findings highlight the potential utility of EEG as a non-invasive biomarker for early detection and monitoring of NCDs.

In the present study, we observed that individuals with NCDs have a significant dysbiosis in their gut microbiota, characterized by a reduction in the prevalence of *Kiritimatiellaeota* and *Lentisphaerae*, and a decline in microbial diversity at the phylum, genus, and species levels. These findings are in line with previous research reporting alterations in the gut microbiota of individuals with neurological disorders, such as AD and PD [43, 44]. For instance, previous studies have reported that AD patients have a significantly lower abundance of *Firmicutes* and higher abundance of *Bacteroidetes* compared to healthy controls [45, 46]. In our own investigation, we observed a similar trend in the NCDs group, with a lower F/B ratio. While this trend was consistent with prior research, this difference did not reach statistical significance. This may be attributed to the specific characteristics of our study population or other factors influencing the gut microbiota composition. Moreover, the decrease in microbiota diversity indices observed in our study aligns with similar reductions reported in individuals with AD [47]. Our study further revealed a significant decrease in the abundance of predicted pathways related to the biosynthesis of aromatic amino acids and the TCA cycle in individuals with NCDs, suggesting that alterations in gut microbial composition may disrupt key metabolic pathways that are important for maintaining cognitive function. Notably, several bacterial taxa identified by LEfSe analysis as specific for NCDs have been previously reported to be associated with AD, including *Porphyromonas asaccharolytica* and *Ruminococcus gnavus* [47, 48]. As for the different characteristic species, it is important to consider the impact of environmental factors, such as diet, medication use, and geographical location, which can influence the composition of gut microbiota and may contribute to variations in results across different studies.

In line with prior research, our study has demonstrated that modifications in protein expression may exert an influence on the metabolism of amino acids and the production of energy, thereby potentially exacerbating the onset and progression of NCDs [49, 50]. Previous studies provide evidence of a correlation between cognitive decline and abnormal proteins expression, suggesting a potential role for these changes in the pathogenesis of NCDs [51, 52]. Perturbations in protein expression patterns can lead to imbalances in the levels of amino acids, including glutamate, which has been linked to neurotoxicity and cognitive dysfunction. Additionally, alterations in protein expression may impair energy production pathways, such as mitochondrial function, which are frequently compromised in the context of NCDs. Proteomics analysis has emerged as a powerful tool for investigating proteins expression changes in NCDs, with several studies using this approach to identify potential biomarkers for early diagnosis and therapeutic targets for cognitive impairment [53]. In particular, proteomics analysis of cerebrospinal fluid (CSF) has been employed to identify differentially expressed proteins in Alzheimer’s disease (AD) and HIV-associated neurocognitive disorders (HAND) [54, 55]. However, our study revealed unique findings in addition to those reported in prior studies. For example, we identified several KEGG pathways associated with inflammatory bowel disease, bladder cancer, type I diabetes mellitus, and long-term depression in the upregulated proteins of the NCDs group. Although these pathways have not been extensively studied in NCDs, some studies have suggested potential links between them [56, 57]. Therefore, the identification of these pathways may provide new insights into the pathogenesis of NCDs and could be useful for developing new therapeutic strategies. It is worth noting that the significance of a pathway represented by a single protein may not necessarily reflect its true functional relevance within the broader biological context. Therefore, the potential functional importance of these pathways should be further validated through additional experimental and functional studies.

This study presents additional evidence for the complex interplay between metabolites and NCDs. Specifically, numerous metabolites have been identified as playing crucial roles in the development of NCDs, including NAD + , L-tryptophan, ursodeoxycholic acid, L-kynurenine, L-glutamic acid, nicotinic acid, GABA, and nervonic acid. For example, a decrease in NAD + levels, an essential coenzyme in cellular metabolism, has been associated with aging and neurodegenerative diseases [58]. Similarly, L-tryptophan, a precursor to serotonin, has been linked to mood disorders such as depression and anxiety [59]. In addition, ursodeoxycholic acid, a kind of bile acid, has been demonstrated to have potential therapeutic effects in Alzheimer’s disease (AD) by correcting mitochondrial morphology and membrane structure through its impact on dynamin-related protein 1 [60]. L-Kynurenine, a metabolite of tryptophan, has been implicated in neuroinflammation and neurodegeneration [61], while dysregulation of L-glutamic acid, a crucial neurotransmitter in the central nervous system, has been linked to the development of neurodegenerative diseases [62]. Moreover, nicotinic acid, a form of vitamin B3, has shown neuroprotective properties and is associated with cognitive function [63]. GABA, another crucial neurotransmitter, has been linked to various physiological processes in the brain, including mood disorders [64], while nervonic acid, an omega-9 fatty acid, has been associated with cognitive function [65]. Although there is a considerable body of evidence in the literature supporting the role of these metabolites in neurocognitive disorders, the underlying mechanisms require further investigation. Furthermore, a more extensive examination of the roles of L-kynurenine, L-glutamic acid, nicotinic acid, GABA, and nervonic acid in neurocognitive disorders is needed to provide a comprehensive understanding of the complex interplay between metabolites and NCDs. Remarkably, FAHFAs represent a newly discovered class of lipids that have implicated in various physiological processes, including metabolism, inflammation, and insulin sensitivity. Studies have suggested that alterations in FAHFA levels may be associated with neurocognitive disorders. For example, a decline in FAHFAs in adipose tissue of individuals with obesity and insulin resistance indicates a potential action on metabolic dysfunction [66]. Similarly, the correlation between decreased FAHFAs and inflammation has been found in patients with acute coronary syndrome. Furthermore, the identification of specific metabolic pathways associated with neurocognitive disorders, such as nicotinate and nicotinamide metabolism, suggests a potential link between FAHFAs and NAD + metabolism, which has been shown to play a role in cognitive function [67]. The association between FAHFAs and cognitive function warrants further investigation and may provide new insights into the underlying mechanisms of NCDs.

The present study also assessed the impact of FMT on the gut microbiota and associated metabolites. Bacterial biomarkers for NCDs were identified, and the relative abundance of *Ruminococcus gnavus* and *Lachnoclostridium* sp. *YL 32* increased significantly in mice, consistent with population-level changes. Metabolic pathways predicted by metagenome sequencing, including the biosynthesis of aromatic amino acids and the TCA cycle, were validated through analysis of serum and hippocampal metabolomics. Significant changes in the relative content of citric acid, fumaric acid, succinic acid, oxoglutaric acid, pyruvic acid, and tryptophan were observed. The biosynthesis of aromatic amino acids is a vital metabolic process that generates three essential amino acids, including tryptophan, which is necessary for protein synthesis, neurotransmitter synthesis, and immune regulation [68]. Previous studies have reported the alterations in biosynthesis of aromatic amino acids in cognitive impairment, with significant decreases in tryptophan concentration observed in patients with AD and MCI [69, 70]. The present study found significant changes in the relative content of tryptophan in both hippocampal and serum samples of NCD-FMT mice, suggesting a potential link between alterations in the biosynthesis of aromatic amino acids and cognitive impairment in NCDs. The TCA cycle is a central metabolic pathway that plays a crucial role in cellular energy production through the oxidation of acetyl-CoA derived from carbohydrates, fatty acids, and amino acids. The TCA cycle generates ATP, which is necessary for various cellular processes, including neurotransmitter synthesis, ion channel transport, and axonal growth [71–73]. The dysregulation of these pathways has been implicated in the pathogenesis and progression of NCDs, with previous studies reporting impaired TCA cycle activity in patients with AD and the 5xFAD mouse model of AD [74, 75].

From the correlation analysis, we have found that EEG oscillation was correlated with the diversity of gut microbiota and metabolomics. Here, the EEG, recording electrical activity of the brain, has been used to investigate the potential relationship between gut microbiota and brain function. There is growing evidence to suggest that the gut microbiota, which is the collection of microorganisms that live in the digestive tract, may have an impact on brain function and behavior [76]. The gut and brain communicate bidirectionally through several pathways, including the vagus nerve, immune system, and production of neurotransmitters and hormones [76]. Several studies have suggested that the composition and diversity of gut microbiota may influence EEG activity. For example, one study found that mice with a disrupted microbiome had altered EEG patterns compared to control mice, suggesting that the gut microbiota may play a role in regulating brain activity [77]. Another study found that individuals with higher levels of diversity in their gut microbiota had a more stable and balanced EEG pattern, while those with lower diversity had a more erratic EEG pattern [78]. Research has also suggested that specific strains of bacteria may have an impact on brain function and EEG activity. For example, *Lactobacillus rhamnosus* has been shown to improve anxiety- and depression-like behaviors in mice, and to increase GABA receptor expression in the brain, which is associated with calming effects [79]. GABA receptor expression has also been linked to EEG activity, suggesting a potential mechanism by which gut bacteria may influence brain function [80]. Notably, both glutamate and GABA exert effects on microglial cells, impacting their activation, inflammatory responses, and clearance of Aβ [81]. In AD, microglia express various glutamate and GABA receptors, including NMDA, AMPA, KA, mGluR2-8, and GABAA/B [82]. Aβ further disrupts glutamatergic signaling, activating microglial cells through multiple receptors, leading to neuroinflammation and oxidative stress [83]. Overall, while more research is needed to fully understand the relationship between gut microbiota and EEG activity, there is growing evidence to suggest that the two are linked. This could have important implications for the development of therapies targeting the gut-brain axis for neurological and psychiatric disorders.

### Limitation and clinical perspectives

One of the primary limitations of this study is the relatively small sample size. The analysis and machine learning model development were conducted on a matched cohort of NA and NCDs. While the results are promising, the small sample size may limit the generalizability of the findings to larger and more diverse populations. Another limitation is the absence of an external validation dataset. The machine learning model’s performance was evaluated using cross-validation techniques, which are valuable for assessing internal consistency. However, external validation on an independent dataset from a different source or population is essential to confirm the model’s real-world applicability and generalizability. Omics data often involve the simultaneous testing of thousands or even millions of variables (e.g., genes, metabolites, proteins). This mass testing can result in an increased likelihood of false positives purely due to the large number of comparisons made, leading to an elevated type I error rate. It is crucial to consider the practical significance and effect size in addition to statistical significance.

In spite of these limitations, this study presents substantial clinical prospects. It offers the potential for a more personalized and precise diagnostic strategy, enabling more effective longitudinal treatment evaluation and the exploration of innovative neuromodulation techniques. These advancements hold promise for enhancing our understanding and management of NCDs.

## Conclusions

In this study, we conducted an integrated analysis of EEG, microbial, proteomic, and metabolomic data from both normal aging seniors and those with NCDs. Our analysis revealed several key findings. First, we observed decreased EEG power spectral density (PSD). Second, we identified disturbances in the biosynthesis of aromatic amino acids and the tricarboxylic acid cycle. These disturbances were associated with increased levels of specific microbes, including *Ruminococcus gnavus*, *Enterocloster bolteae*, and *Lachnoclostridium* sp. *YL 32*. Additionally, we found decreased levels of specific metabolites, such as L-tryptophan, L-glutamic acid, γ-aminobutyric acid (GABA), and FAHFAs. These findings collectively provide insights into potential features of NCDs pathophysiology. Moreover, it suggests that the application of the machine learning model greatly improve the distinguishing degree of NCDs in the participants. Therefore, the fusion of multi-omics profiles and EEG data holds particular promise for personalized diagnostics in NCDs patients.

### Supplementary Information

**Additional file 1: Supplementary Data 1.** The EEG power spectral density and EEG microstate of seniors in normal aging and neurocognitive disorders group.

**Additional file 2: Supplementary Data 2. **The taxonomic annotation of gut microbiota of seniors in normal aging and neurocognitive disorders group.

**Additional file 3: Supplementary Data 3. **The predictive bacterial functional profiles of BioCyc pathways of seniors in normal aging and neurocognitive disorders group.

**Additional file 4: Supplementary Data 4. **The quantitative proteomics profiles of urine exosomes of seniors in normal aging and neurocognitive disorders group.

**Additional file 5: Supplementary Data 5. **The metabolites quantification profiles in positive ion modes of seniors in normal aging and neurocognitive disorders group.

**Additional file 6: Supplementary Data 6.**The metabolites quantification profiles in negative ion modes of seniors in normal aging and neurocognitive disorders group.

**Additional file 7: Supplementary Data 7.** The taxonomic annotation of gut microbiota of mice at different time points.

**Additional file 8: Supplementary Data 8.**The taxonomic annotation of gut microbiota of mice in control, NA-FMT, and NCDs-FMT group.

**Additional file 9: Supplementary Data 9.**The metabolomic analysis profiles of serum in fecal microbiota transplantation mice.

**Additional file 10: Supplementary Data 10. **The metabolomic analysis profiles of hippocampus in fecal microbiota transplantation mice.

**Additional file 11. **Supplementary Results. **Supplementary Figure S1. **Gut microbiota diversity analysis in normal aging and neurocognitive disorders (NCDs) groups. Alpha diversity analysis were measured by Chao1 index, Shannon index, and Simpson index at the species level. Beta diversity analysis was measured by Bray-Curtis dissimilarity, with P values computed using a two-sided Wilcoxon test. **Supplementary Figure S2. **Identification and characterization of urinary exosomes. (A) Morphological characteristics of exosomes observed by transmission electron microscopy (TEM). (B) The size of the exosomes was determined using nanoparticle tracking analysis (NTA). (C) Marker protein expression of exosomes detected by Western blot. **Supplementary Figure S3.** The contrast ROC curves of different omics discriminated NA and NCDs via leave one out cross validation. The contrast ROC of single omics (Panel A). The contrast ROC of double omics (Panel B). The ROC of multi-omics (Panel C). The EEG related ROC (Panel D). The gut microbiota related ROC (Panel E). The metabolomic related ROC (Panel F). Meta, metabolomic; GM, gut microbiota. **Supplementary Figure S4.** The contrast PR-ROC curves of different omics discriminated NA and NCDs via leave one out cross validation. The contrast ROC of single omics (Panel A). The contrast ROC of double omics (Panel B). The ROC of multi-omics (Panel C). The EEG related ROC (Panel D). The gut microbiota related ROC (Panel E). The metabolomic related ROC (Panel F). Meta, metabolomic; GM, gut microbiota. **Supplementary Table S1.** The machine learning performance of different omics via 10-fold cross-validation. The values were presented as mean (standard deviation). **Supplementary Figure S5.** The ROC curves of different omics via 10-fold cross-validation. The single omics discriminated the normal aging (NA) and neurocognitive disorders (NCDs) group in blue (Panel A-C). The double omics discriminated the NA and NCDs group in green (Panel D-F). The classification of multi-omics (Panel G). Meta, metabolomic; GM, gut microbiota. **Supplementary Figure S6.** The contrast ROC curves of different omics discriminated NA and NCDs via 10-fold cross-validation. The contrast ROC of single omics (Panel A). The contrast ROC of double omics (Panel B). The ROC of multi-omics (Panel C). The EEG related ROC (Panel D). The gut microbiota related ROC (Panel E). The metabolomic related ROC (Panel F). Meta, metabolomic; GM, gut microbiota. **Supplementary Figure S7.** The PR-ROC of different omics via 10-fold cross-validation. The single omics discriminated the NA and NCDs group in blue (Panel A-C). The double omics discriminated the NA and NCDs group in green (Panel D-F). The classification of multi-omics (Panel G). Meta, metabolomic; GM, gut microbiota. **Supplementary Figure S8.** The contrast PR-ROC of different omics discriminated NA and NCDs via 10-fold cross-validation. The contrast PR-ROC of single omics (Panel A). The contrast PR-ROC of double omics (Panel B). The PR-ROC of multi-omics (Panel C). The EEG related PR-ROC (Panel D). The gut microbiota related PR-ROC (Panel E). The metabolomic related PR-ROC (Panel F). Meta, metabolomic; GM, gut microbiota. **Supplementary Table S2.** The machine learning performance of different omics via 5-fold cross-validation. **Supplementary Figure S9.** The ROC of different omics via 5-fold cross-validation. The single omics discriminated the NA and NCDs group in blue (Panel A-C). The double omics discriminated the NA and NCDs group in green (Panel D-F). The classification of multi-omics (Panel G). Meta, metabolomic; GM, gut microbiota. **Supplementary Figure S10.** The contrast ROC of different omics discriminated NA and NCDs via 5-fold cross-validation. The contrast ROC of single omics (Panel A). The contrast ROC of double omics (Panel B). The ROC of multi-omics (Panel C). The EEG related ROC (Panel D). The gut microbiota related ROC (Panel E). The metabolomic related ROC (Panel F). Meta, metabolomic; GM, gut microbiota. **Supplementary Figure S11.** The PR-ROC of different omics via 5-fold cross-validation. The single omics discriminated the NA and NCDs group in blue (Panel A-C). The double omics discriminated the NA and NCDs group in green (Panel D-F). The classification of multi-omics (Panel G). Meta, metabolomic; GM, gut microbiota. **Supplementary Figure S12.** The contrast PR-ROC of different omics discriminated NA and NCDs via 5-fold cross-validation. The contrast PR-ROC of single omics (Panel A). The contrast PR-ROC of double omics (Panel B). The PR-ROC of multi-omics (Panel C). The EEG related PR-ROC (Panel D). The gut microbiota related PR-ROC (Panel E). The metabolomic related PR-ROC (Panel F). Meta, metabolomic; GM, gut microbiota. **Supplementary Figure S13.** The selected features. Panel A) The left is bar plot of selected features and their contributions of EEG, metabolomic and gut microbiota model (weight in SVM) in descending order. The right is a bee swarm plot in which each point represents a participant (n = 41). Panel B) Feature category contribution calculated by summing the weight in EEG, metabolomic and gut microbiota model. Panel C and panel D represented the gut microbiota and metabolomics respectively. In bar plot, blue bar represented higher value of the feature for association with NCDs while the red bar presented higher association with normal aging. In bee swarm plot, color indicates the value of the feature, with red higher and blue lower. Negative contribution indicates the feature attribution for prediction of NCDs while Positive contribution indicates the feature attribution of normal aging. Meta, metabolomic; GM, gut microbiota. 

## Data Availability

The data that support the findings of this study are available from the corresponding author upon reasonable request.

## Abbreviations

NCDs: Neurocognitive disorders
EEG: Electroencephalography
NA: Normal aging
FMT: Faecal microbiota transplantation
GABA: Gamma-aminobutyric acid
FAHFAs: Fatty acid esters of hydroxy fatty acids
SVM: Support vector machine
SCFAs: Short chain fatty acids
MoCA: Montreal Cognitive Assessment
ICA: Independent component analysis
PSD: Power spectral density
GFP: Global field power
TEM: Transmission electron microscope
NTA: Nanoparticle tracking analysis
HUMAnN2: HMP Unified Metabolic Analysis Network
PCoA: Principal coordinate analysis
PCA: Principal component analysis
LEfSe: Linear discriminant analysis effective size
PLS-DA: Partial least squares discriminant analysis
LOOCV: Leave-one-out cross-validation
FDR: False discovery rate
