# Supplementary material for: The fusion of multi-omics profile and multimodal EEG data contributes to the personalized diagnostic strategy for neurocognitive disorders
Source: Microbiome. 2024 Jan 19;12:12. doi: 10.1186/s40168-023-01717-5 (PMC10797890; doi:10.1186/s40168-023-01717-5)
Supplement: Supplementary file 12 — Additional file 11. Supplementary Results. Supplementary Figure S1. Gut microbiota diversity analysis in normal aging and neurocognitive disorders (NCDs) groups. Alpha diversity analysis were measured by Chao1 index, Shannon index, and Simpson index at the species level. Beta diversity analysis was measured by Bray-Curtis dissimilarity, with P values computed using a two-sided Wilcoxon test. Supplementary Figure S2. Identification and characterization of urinary exosomes. (A) Morphological characteristics of exosomes observed by transmission electron microscopy (TEM). (B) The size of the exosomes was determined using nanoparticle tracking analysis (NTA). (C) Marker protein expression of exosomes detected by Western blot. Supplementary Figure S3. The contrast ROC curves of different omics discriminated NA and NCDs via leave one out cross validation. The contrast ROC of single omics (Panel A). The contrast ROC of double omics (Panel B). The ROC of multi-omics (Panel C). The EEG related ROC (Panel D). The gut microbiota related ROC (Panel E). The metabolomic related ROC (Panel F). Meta, metabolomic; GM, gut microbiota. Supplementary Figure S4. The contrast PR-ROC curves of different omics discriminated NA and NCDs via leave one out cross validation. The contrast ROC of single omics (Panel A). The contrast ROC of double omics (Panel B). The ROC of multi-omics (Panel C). The EEG related ROC (Panel D). The gut microbiota related ROC (Panel E). The metabolomic related ROC (Panel F). Meta, metabolomic; GM, gut microbiota. Supplementary Table S1. The machine learning performance of different omics via 10-fold cross-validation. The values were presented as mean (standard deviation). Supplementary Figure S5. The ROC curves of different omics via 10-fold cross-validation. The single omics discriminated the normal aging (NA) and neurocognitive disorders (NCDs) group in blue (Panel A-C). The double omics discriminated the NA and NCDs group in green (Panel D-F). The classification [file 40168_2023_1717_MOESM11_ESM.docx]

**Supplementary Results**

**Results**

**Machine learning classification**

To mitigate the potential influence of cross-validation, we employed Support Vector Machines (SVMs) to construct machine learning models using both 5-fold and 10-fold cross-validation techniques. These models aimed to classify individuals into either the normal aging or neurocognitive disorders (NCDs) groups based on features derived from three distinct data sources: omics (metabolomic and gut microbiota) and electroencephalography (EEG). As presented in Table 3 and supplementary Tables S1-2, the classification performance consistently demonstrated robustness across various cross-validation methods, including Leave-One-Out Cross-Validation (LOOCV), 5-fold cross-validation, and 10-fold cross-validation. For the 5-fold cross-validation and 10-fold cross-validation, we have conducted statistic to compare their difference across modalities. Within the scope of single omics data, the SVM-based classification performance, when using metabolomic features, exhibited superior discriminatory power compared to both EEG and gut microbiota data (AUC _metabolomic_ > AUC _EEG_ > AUC _Gut microbiota_, *p* < 0.001). When considering two omics data sources, SVM-based classification using combined metabolomic and EEG data, as well as combined gut microbiota and metabolomic data, outperformed the model combining EEG and gut microbiota data (AUC _metabolomic+gut microbiota_, AUC _metabolomic+EEG_ > AUC _EEG+gut microbiota_, *p* < 0.001), with no significant difference observed between models of metabolomic combining gut microbiota and EEG data (*p* = 0.07). Furthermore, we investigated the performance differences among EEG, gut microbiota, and metabolomic-related models. In EEG-related machine learning models, the multi-omics model demonstrated higher performance than the EEG-only model. Additionally, the model combining EEG and metabolomic data outperformed the EEG-only model as well as the EEG and gut microbiota combined model (AUC _multi-omics_ > AUC _metabolomic+EEG_ > AUC _EEG_, AUC _EEG+Gut microbiota_, *p* < 0.001), while no significant difference between EEG-only model and the EEG and gut microbiota combined model (*p* =0.89). In the context of gut microbiota-related machine learning models, the multi-omics model exhibited superior performance to models combining gut microbiota with metabolomic data. The model combining gut microbiota and metabolomic data demonstrated better performance than the gut microbiota combined with EEG model, which, in turn, outperformed the gut microbiota-only model (AUC _multi-omics_ > AUC _gut microbiota+metabolomic_ > AUC _gut microbiota+EEG_ > AUC _gut microbiota_, *p* < 0.001). Finally, in the realm of metabolomic-related machine learning models, the multi-omics model displayed superior performance to models combining gut microbiota with metabolomic data. The model combining gut microbiota and metabolomic data also exhibited better performance than the gut microbiota combined with EEG model, which surpassed the gut microbiota-only model (AUC _multi-omics_ > AUC _gut microbiota+metabolomic_ , AUC _metabolomic+EEG_ , AUC _metabolomic_, *p* < 0.001, AUC *gut microbiota+metabolomic* > AUC _metabolomic_, *p* < 0.001, AUC _gut microbiota+metabolomic_ > AUC _metabolomic+EEG_, *p* = 0.0489, AUC _metabolomic+EEG_ > AUC _metabolomic_, p = 0.0432).

**Selected Features**

In the EEG, metabolomic and gut microbiota model (Figure S13, panel A and B), two EEG features (alpha1 power in FPz and beta1 power in CPz), two gut microbiotas features (corynebacterium striatum, alistipes sp. dk3624) and six metabolomic features (biopterin, benzoic acid, HexCer-NS (d18:2/16:1), alistipes sp. dk3624; 1-(1-adamantyl)-2-[(2-chloro-4-fluorophenyl)thio]ethan-1-one, N3,N4-Dimethyl-L-arginine；acetic acid) were identified, which might be the potential biomarkers for NCDs. Meanwhile ten gut microbiota and ten metabolomic data were also identified (Figure S13, panel C and D).

**Supplementary Figure S1.** Gut microbiota diversity analysis in normal aging and neurocognitive disorders (NCDs) groups. Alpha diversity analysis were measured by Chao1 index, Shannon index, and Simpson index at the species level. Beta diversity analysis was measured by Bray-Curtis dissimilarity, with P values computed using a two-sided Wilcoxon test.

**Supplementary Figure S2.** Identification and characterization of urinary exosomes. (A) Morphological characteristics of exosomes observed by transmission electron microscopy (TEM). (B) The size of the exosomes was determined using nanoparticle tracking analysis (NTA). (C) Marker protein expression of exosomes detected by Western blot.


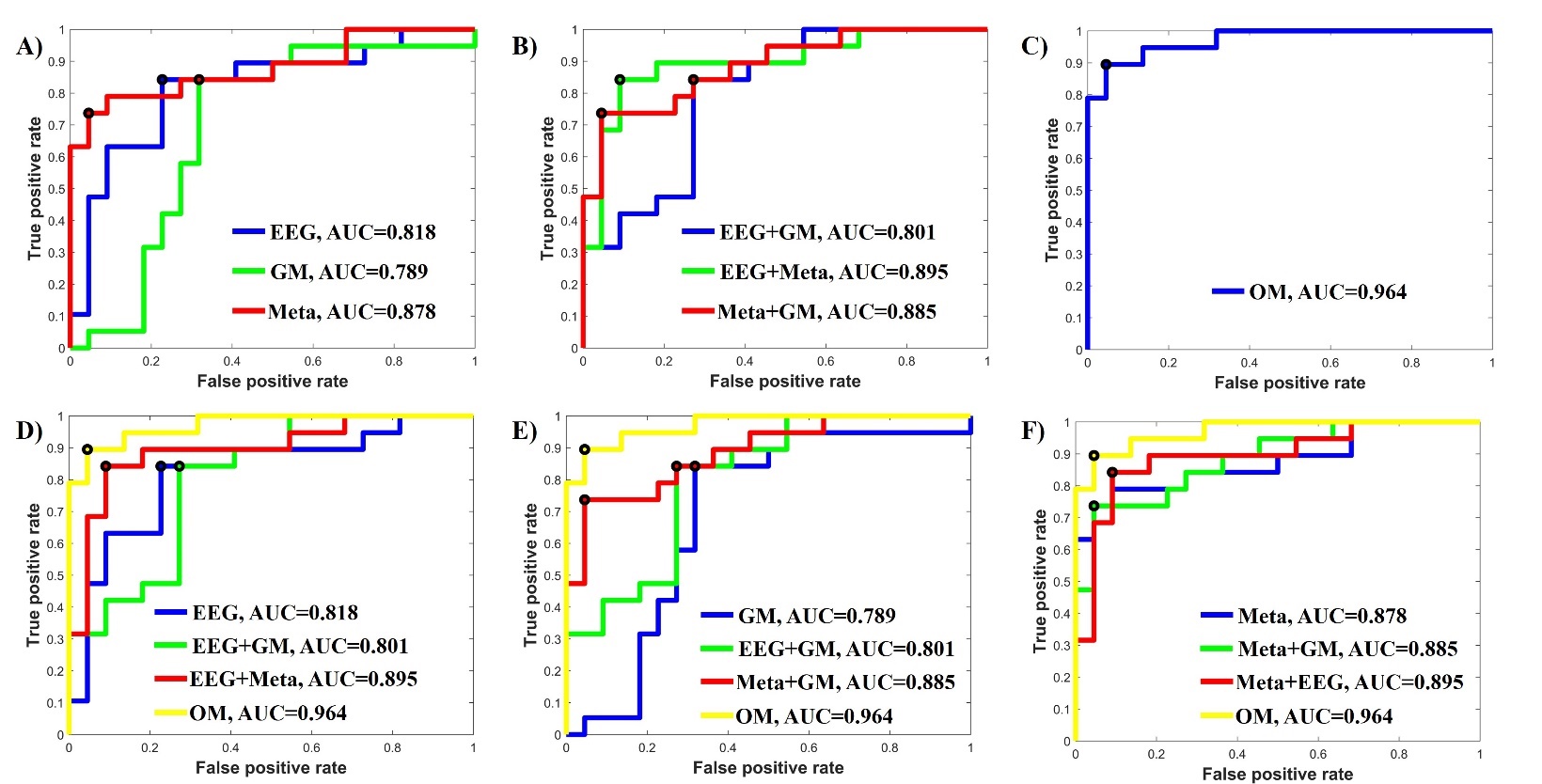


**Supplementary Figure S3.** The contrast ROC curves of different omics discriminated NA and NCDs via leave one out cross validation. The contrast ROC of single omics (Panel A). The contrast ROC of double omics (Panel B). The ROC of multi-omics (Panel C). The EEG related ROC (Panel D). The gut microbiota related ROC (Panel E). The metabolomic related ROC (Panel F). Meta, metabolomic; GM, gut microbiota.


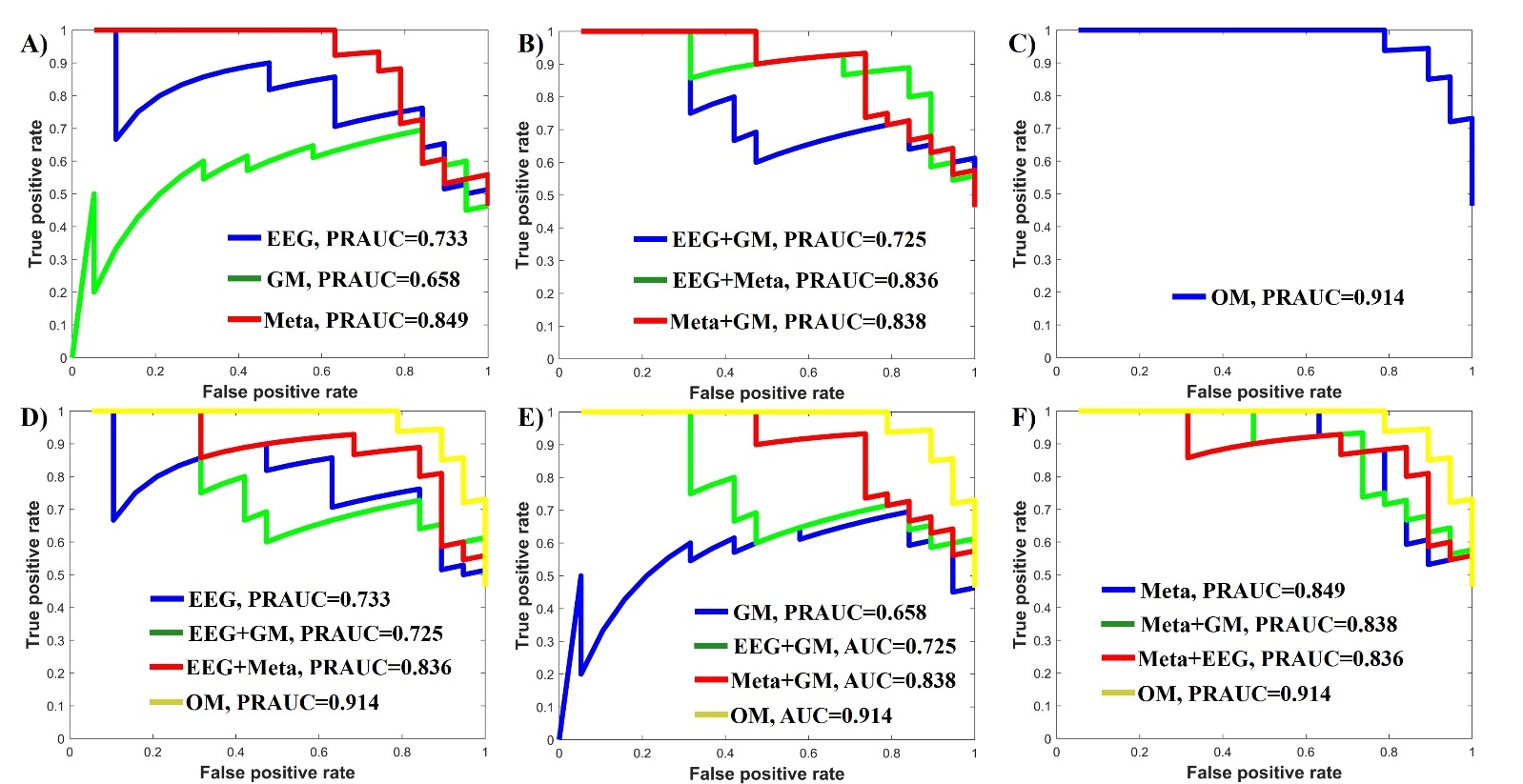


**Supplementary Figure S4.** The contrast PR-ROC curves of different omics discriminated NA and NCDs via leave one out cross validation. The contrast ROC of single omics (Panel A). The contrast ROC of double omics (Panel B). The ROC of multi-omics (Panel C). The EEG related ROC (Panel D). The gut microbiota related ROC (Panel E). The metabolomic related ROC (Panel F). Meta, metabolomic; GM, gut microbiota.

**Supplementary Table S1.** The machine learning performance of different omics via 10-fold cross-validation. The values were presented as mean (standard deviation).

| Modality | ACC | Precise | Recall | F1 score | AUC | PR-AUC | Optimal operating point |
| --- | --- | --- | --- | --- | --- | --- | --- |
| **EEG** | 74.30% (0.0366) | 78.19% (0.0387) | 62.13% (0.0685) | 0.6916 (0.0551) | 0.8044 (0.0230) | 0.7250 (0.0268) | (0.1874 (0.0463), 0.7702 (0.0745)) |
| **Gut Microbiota** | 72.99% (0.0355) | 67.02% (0.0291) | 82.03% (0.0665) | 0.7369 (0.0424) | 0.6904 (0.0217) | 0.5417 (0.0150) | (0. 3415 (0.0354), 0.8678 (0.0613)) |
| **Metabolomic** | 83.82% (0.0242) | 87.94% (0.0399) | 75.61% (0.0348) | 0.8124 (0.0281) | 0.8758 (0.0172) | 0.8444 (0.0134) | (0.0576 (0.0322), 0.7535 (0.0570)) |
| **EEG + Metabolomic** | 84.41% (0.0171) | 86.46% (0.0319) | 78.82% (0.0144) | 0.8243 (0.0175) | 0.8887 (0.0068) | 0.8210 (0.0100) | (0.0632 (0.0335), 0.7985 (0.0454)) |
| **EEG + Gut Microbiota** | 75.07% (0.0296) | 69.49% (0.0290) | 82.54% (0.0380) | 0.7542 (0.0290) | 0.7995 (0.0221) | 0.7219 (0.0255) | (0.2882 (0.0553), 0.8280 (0.0824)) |
| **Metabolomic + Gut Microbiota** | 84.41% (0.0188) | 91.01% (0.0382) | 73.81% (0.0220) | 0.8164 (0.0204) | 0.8986 (0.0167) | 0.8469 (0.0182) | (0.0499 (0.0198), 0.7394 (0.0332)) |
| **EEG + Metabolomic + Gut Microbiota** | 89.83% (0.0249) | 88.92% (0.0461) | 89.47% (0.01) | 0.8913 (0.0235) | 0.9632 (0.0106) | 0.9139 (0.0082) | (0.0366 (0.0182), 0.8845 (0.0316)) |


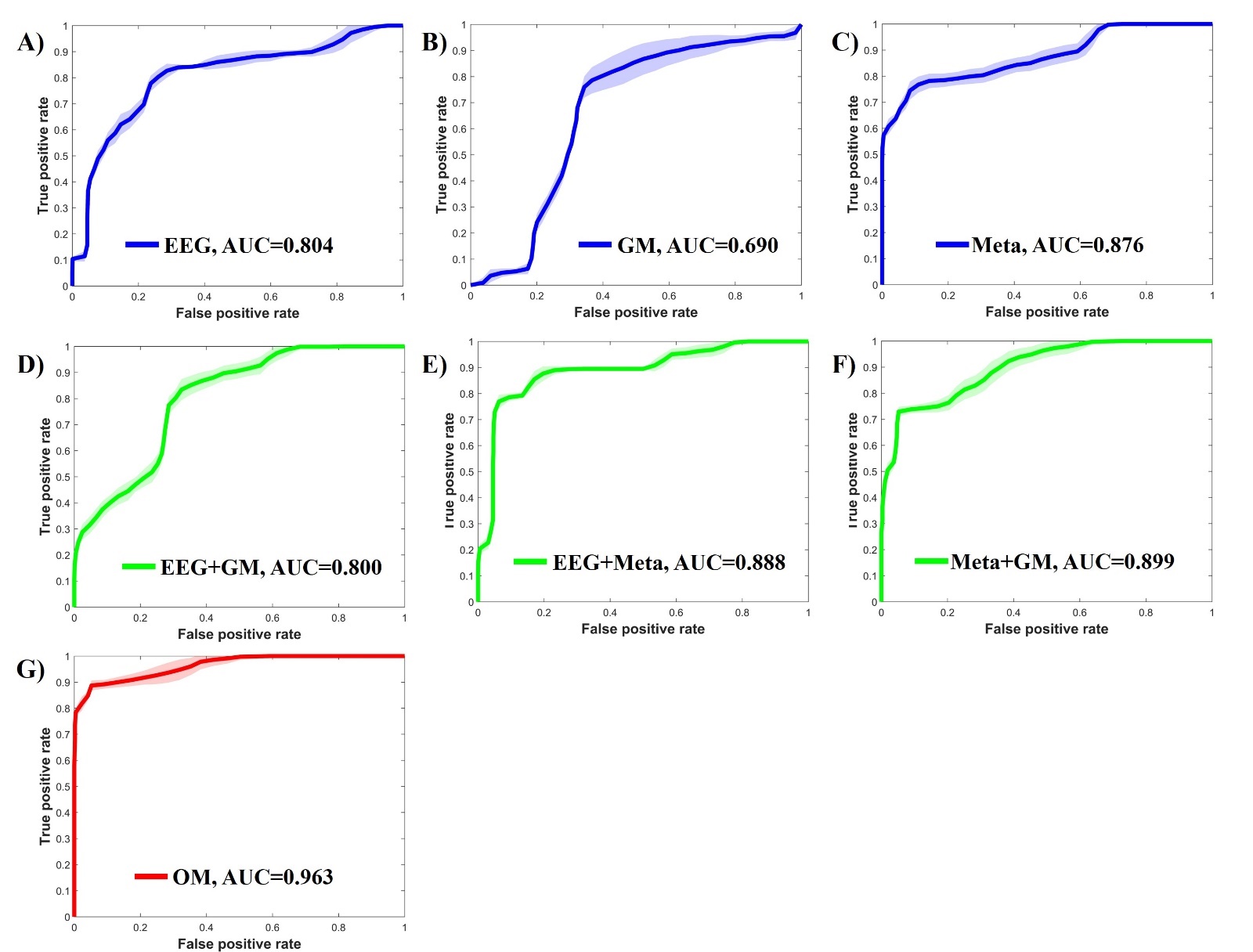


**Supplementary Figure S5.** The ROC curves of different omics via 10-fold cross-validation. The single omics discriminated the normal aging (NA) and neurocognitive disorders (NCDs) group in blue (Panel A-C). The double omics discriminated the NA and NCDs group in green (Panel D-F). The classification of multi-omics (Panel G). Meta, metabolomic; GM, gut microbiota.


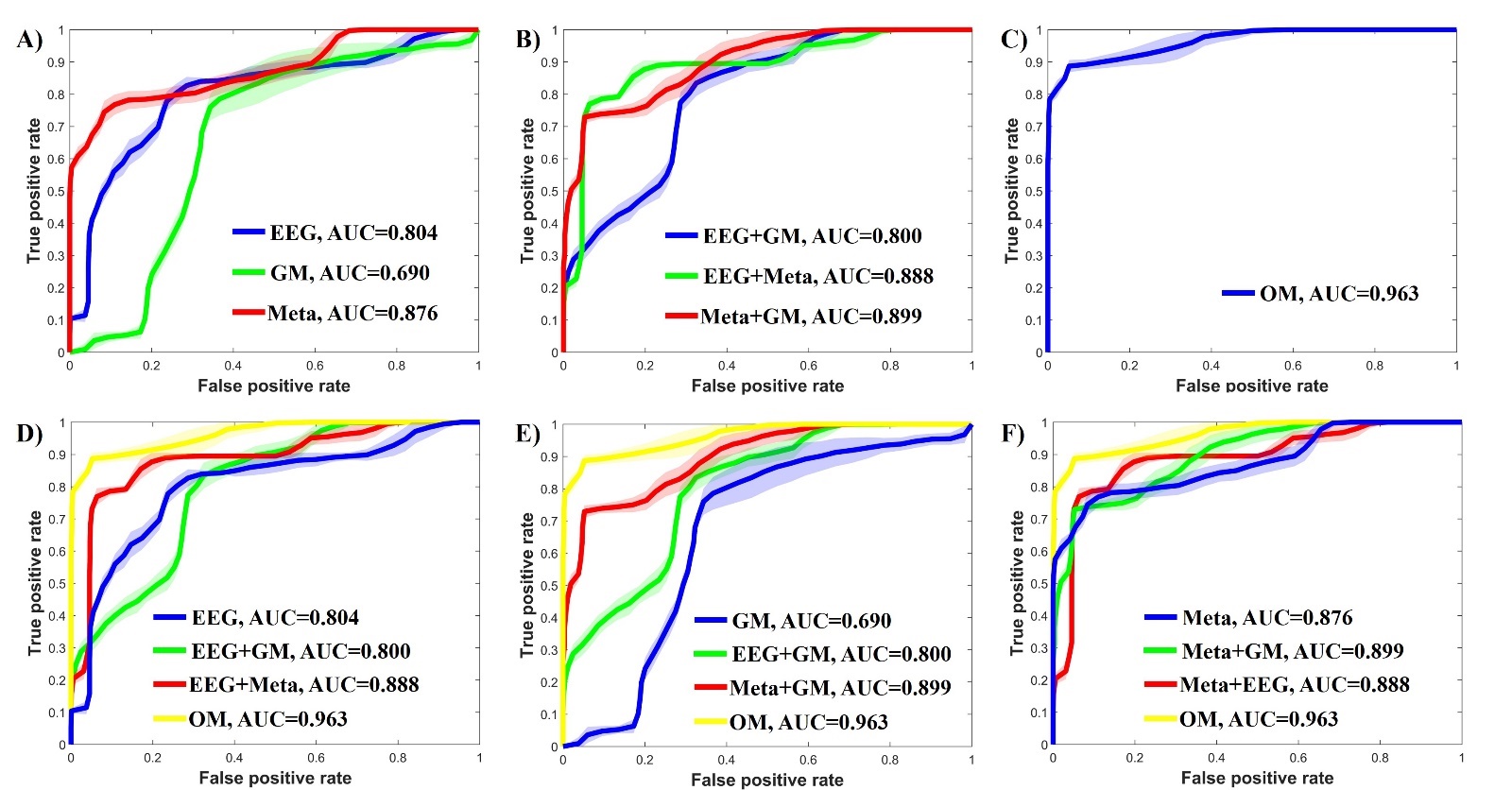


**Supplementary Figure S6.** The contrast ROC curves of different omics discriminated NA and NCDs via 10-fold cross-validation. The contrast ROC of single omics (Panel A). The contrast ROC of double omics (Panel B). The ROC of multi-omics (Panel C). The EEG related ROC (Panel D). The gut microbiota related ROC (Panel E). The metabolomic related ROC (Panel F). Meta, metabolomic; GM, gut microbiota.


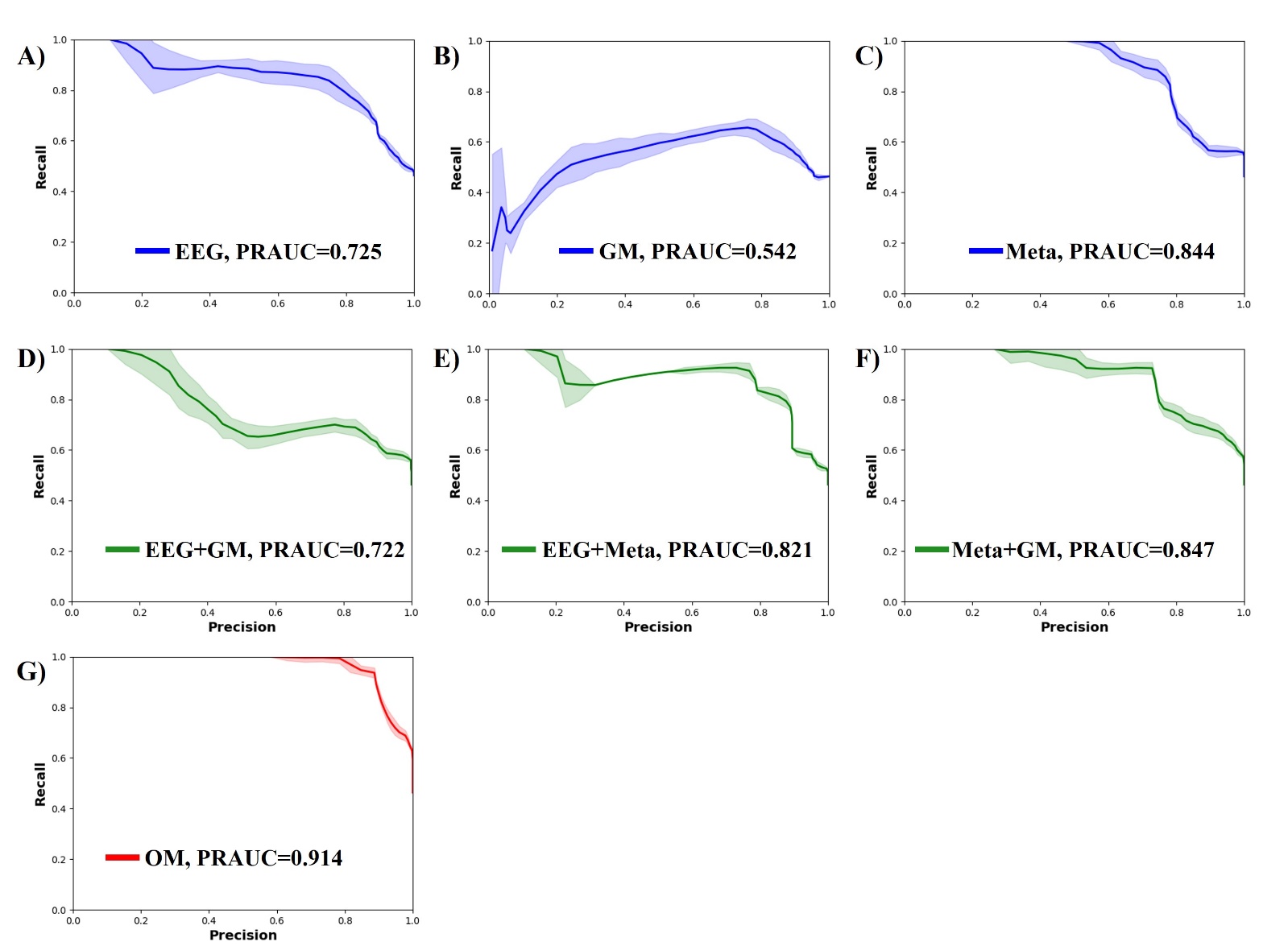


**Supplementary Figure S7.** The PR-ROC of different omics via 10-fold cross-validation. The single omics discriminated the NA and NCDs group in blue (Panel A-C). The double omics discriminated the NA and NCDs group in green (Panel D-F). The classification of multi-omics (Panel G). Meta, metabolomic; GM, gut microbiota.


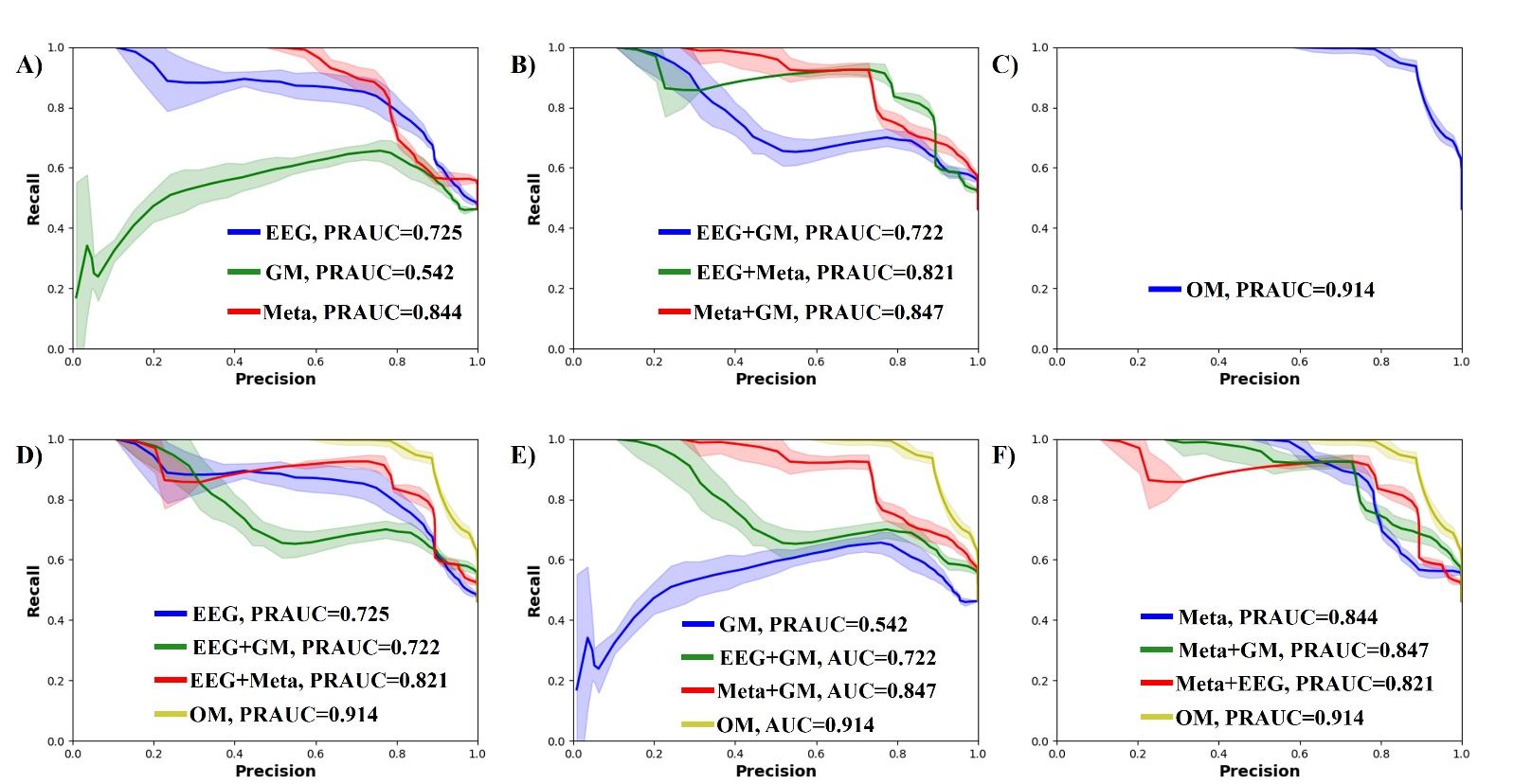


**Supplementary Figure S8.** The contrast PR-ROC of different omics discriminated NA and NCDs via 10-fold cross-validation. The contrast PR-ROC of single omics (Panel A). The contrast PR-ROC of double omics (Panel B). The PR-ROC of multi-omics (Panel C). The EEG related PR-ROC (Panel D). The gut microbiota related PR-ROC (Panel E). The metabolomic related PR-ROC (Panel F). Meta, metabolomic; GM, gut microbiota.

**Supplementary Table S2.** The machine learning performance of different omics via 5-fold cross-validation.

| Modality | ACC | Precise | Recall | F1 score | AUC | PR-AUC | Optimal operating point |
| --- | --- | --- | --- | --- | --- | --- | --- |
| **EEG** | 74.42% (0.0345) | 77.89% (0.0528) | 62.90% (0.0389) | 0.6951 (0.0387) | 0.7988 (0.0312) | 0.7196 (0.0324) | (0.1685 (0.0645), 0.7343 (0.0832)) |
| **Gut Microbiota** | 68.47% (0.0606) | 63.62% (0.0491) | 73.56% (0.1113) | 0.6807 (0.0742) | 0.6662 (0.0482) | 0.5262 (0.0361) | (0. 3736 (0.0686), 0.8472 (0.0871)) |
| **Metabolomic** | 82.75% (0.0444) | 86.39% (0.0711) | 75.10% (0.0486) | 0.8019 (0.0469) | 0.8686 (0.0322) | 0.8373 (0.0252) | (0.0588 (0.0457), 0.7471 (0.0647)) |
| **EEG + Metabolomic** | 84.06% (0.0294) | 86.67% (0.0419) | 77.66% (0.0404) | 0.8185 (0.0345) | 0.8835 (0.0138) | 0.8173 (0.0191) | (0.0765 (0.0502), 0.8036 (0.0486)) |
| **EEG + Gut Microbiota** | 73.41% (0.0411) | 67.88% (0.0391) | 81.26% (0.0612) | 0.7387 (0.0418) | 0.7941 (0.0418) | 0.7149 (0.0542) | (0.3104 (0.099), 0.8357 (0.1213)) |
| **Metabolomic + Gut Microbiota** | 83.64% (0.0257) | 89.01% (0.0471) | 74.07% (0.0298) | 0.8077 (0.0280) | 0.8982 (0.0274) | 0.8442 (0.0327) | (0.0543 (0.0325), 0.7407 (0.0379)) |
| **EEG + Metabolomic + Gut Microbiota** | 88.99% (0.0409) | 86.94% (0.0615) | 90.24% (0.0301) | 0.8846 (0.0396) | 0.9536 (0.0259) | 0.9010 (0.0312) | (0.0443 (0.0552), 0.8793 (0.0658)) |


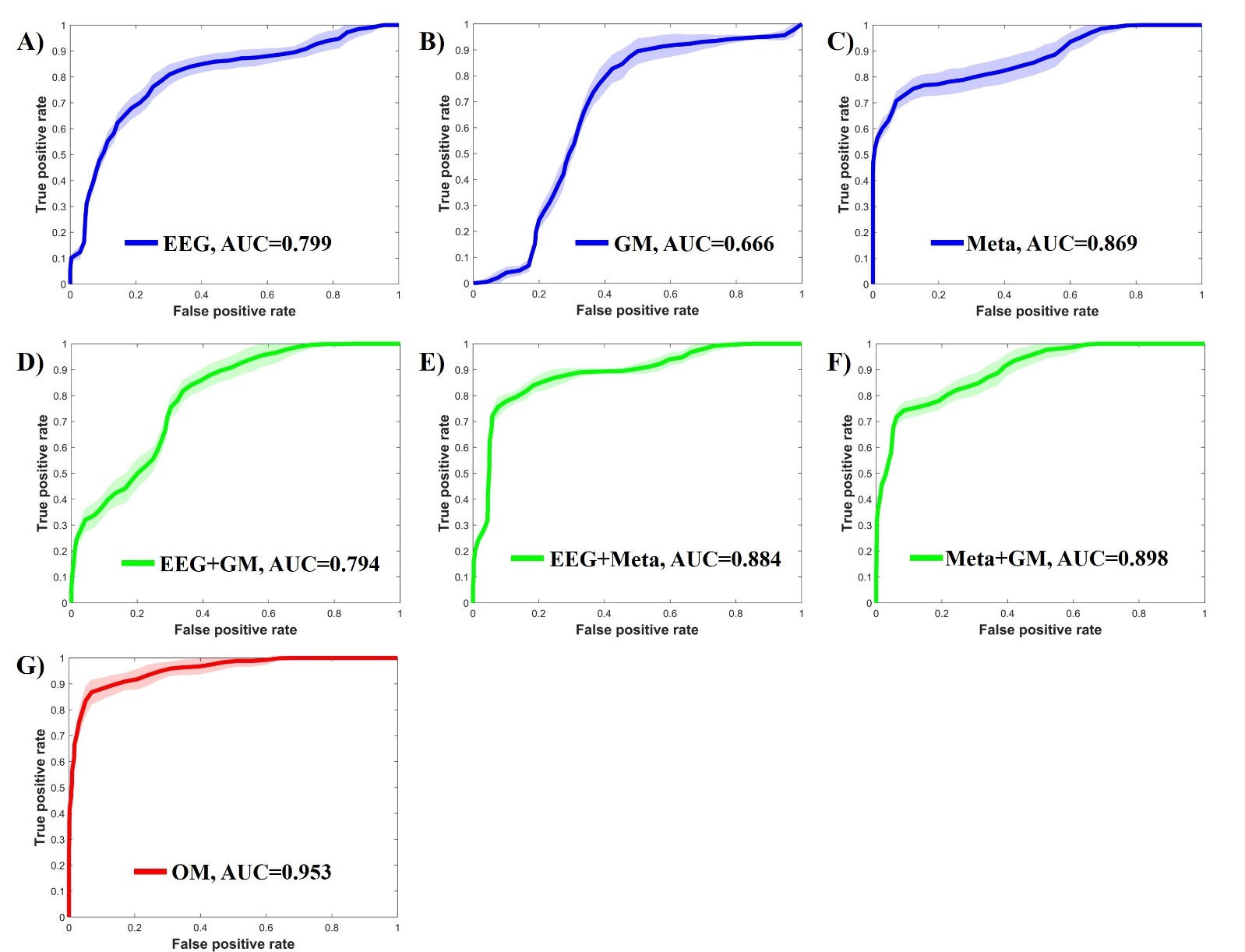


**Supplementary Figure S9.** The ROC of different omics via 5-fold cross-validation. The single omics discriminated the NA and NCDs group in blue (Panel A-C). The double omics discriminated the NA and NCDs group in green (Panel D-F). The classification of multi-omics (Panel G). Meta, metabolomic; GM, gut microbiota.


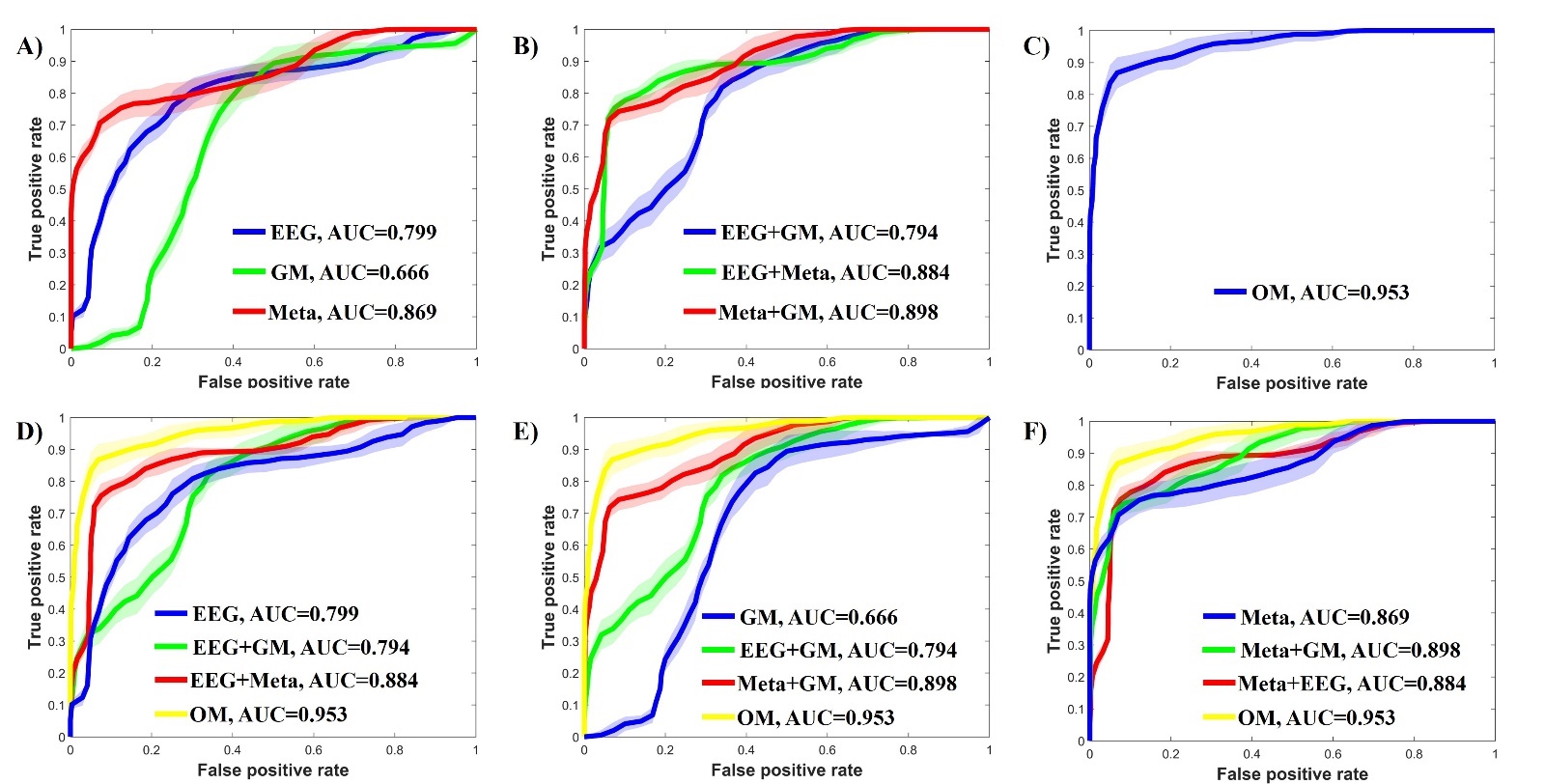


**Supplementary Figure S10.** The contrast ROC of different omics discriminated NA and NCDs via 5-fold cross-validation. The contrast ROC of single omics (Panel A). The contrast ROC of double omics (Panel B). The ROC of multi-omics (Panel C). The EEG related ROC (Panel D). The gut microbiota related ROC (Panel E). The metabolomic related ROC (Panel F). Meta, metabolomic; GM, gut microbiota.


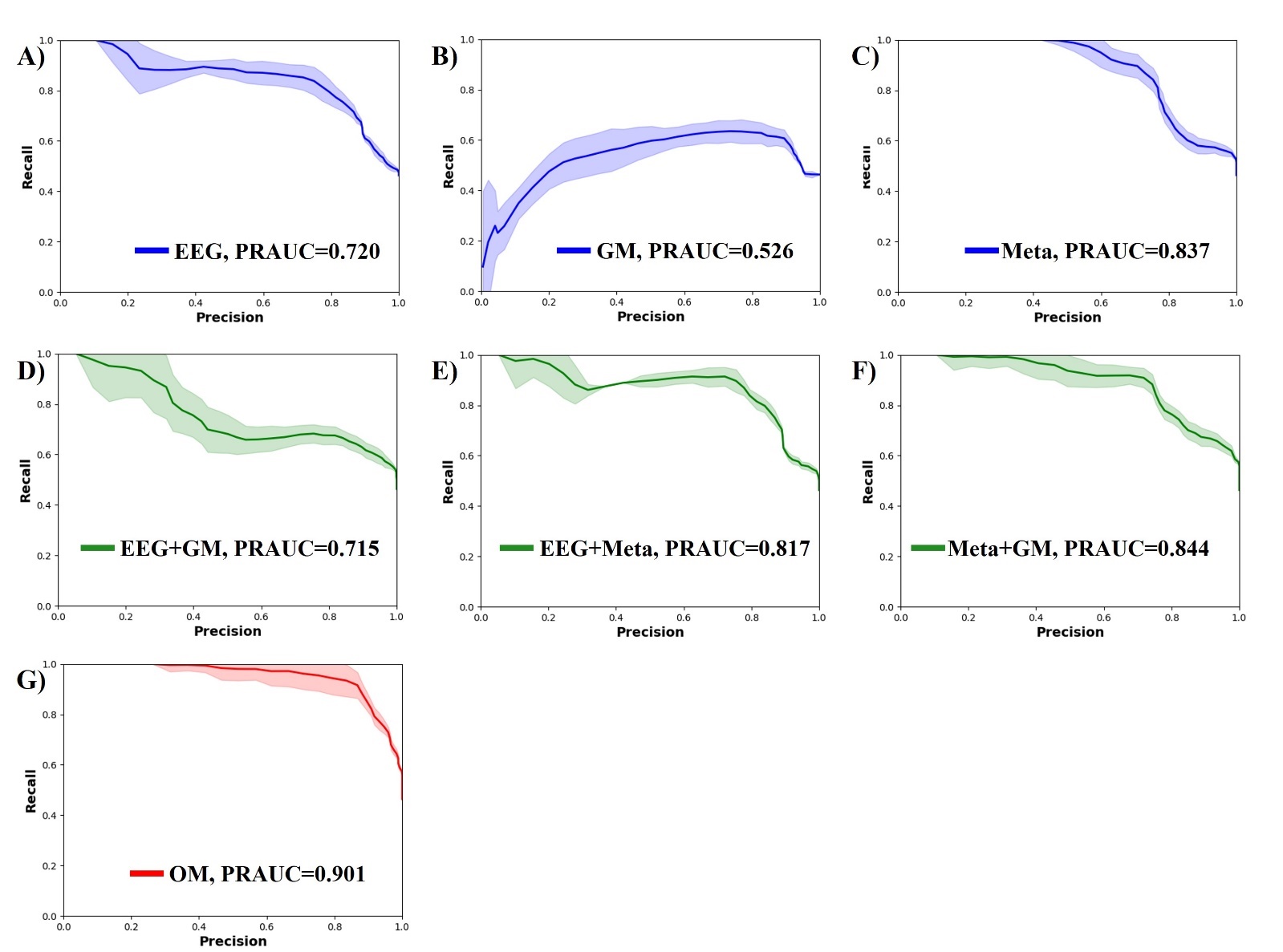


**Supplementary Figure S11.** The PR-ROC of different omics via 5-fold cross-validation. The single omics discriminated the NA and NCDs group in blue (Panel A-C). The double omics discriminated the NA and NCDs group in green (Panel D-F). The classification of multi-omics (Panel G). Meta, metabolomic; GM, gut microbiota.


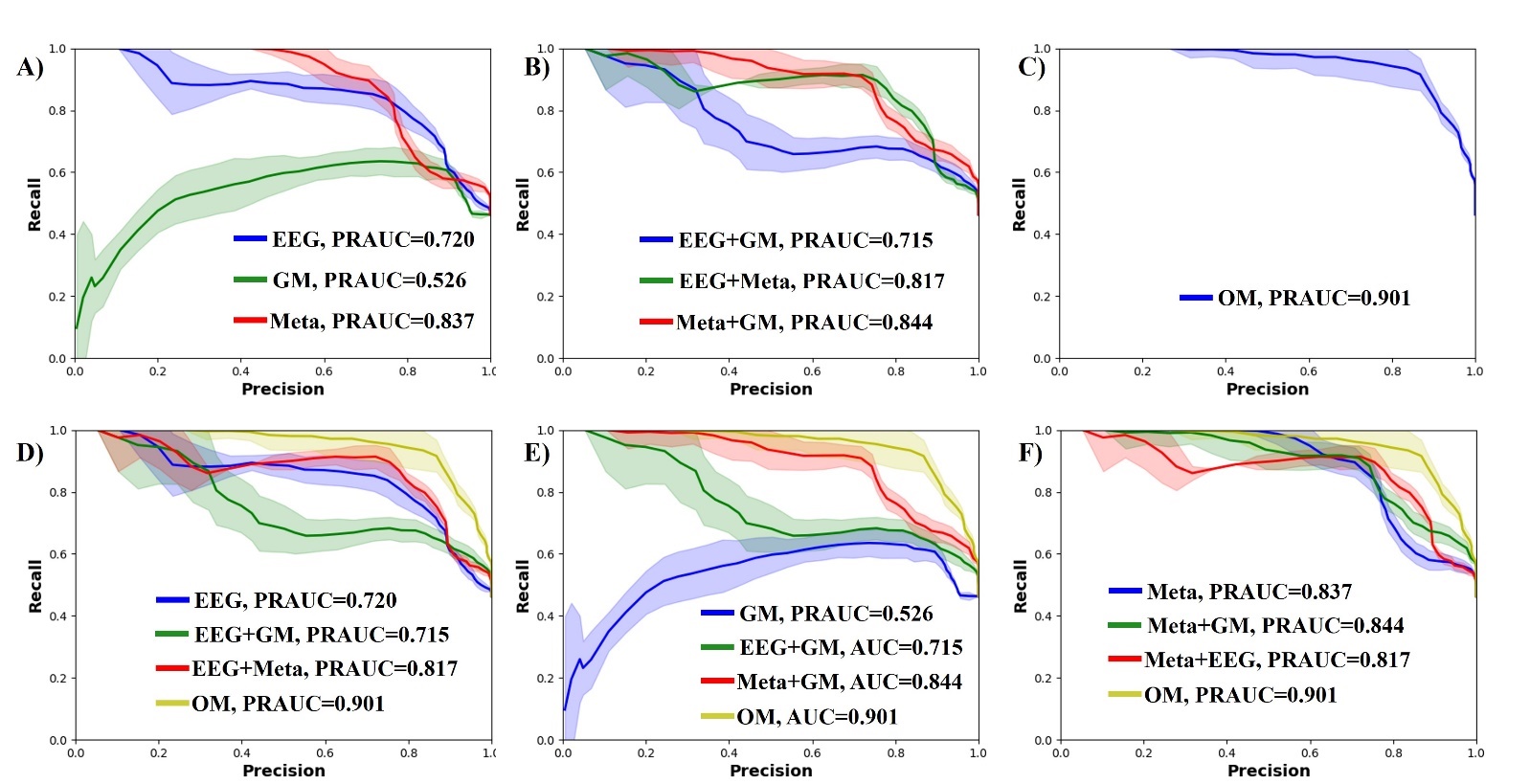


**Supplementary Figure S12.** The contrast PR-ROC of different omics discriminated NA and NCDs via 5-fold cross-validation. The contrast PR-ROC of single omics (Panel A). The contrast PR-ROC of double omics (Panel B). The PR-ROC of multi-omics (Panel C). The EEG related PR-ROC (Panel D). The gut microbiota related PR-ROC (Panel E). The metabolomic related PR-ROC (Panel F). Meta, metabolomic; GM, gut microbiota.


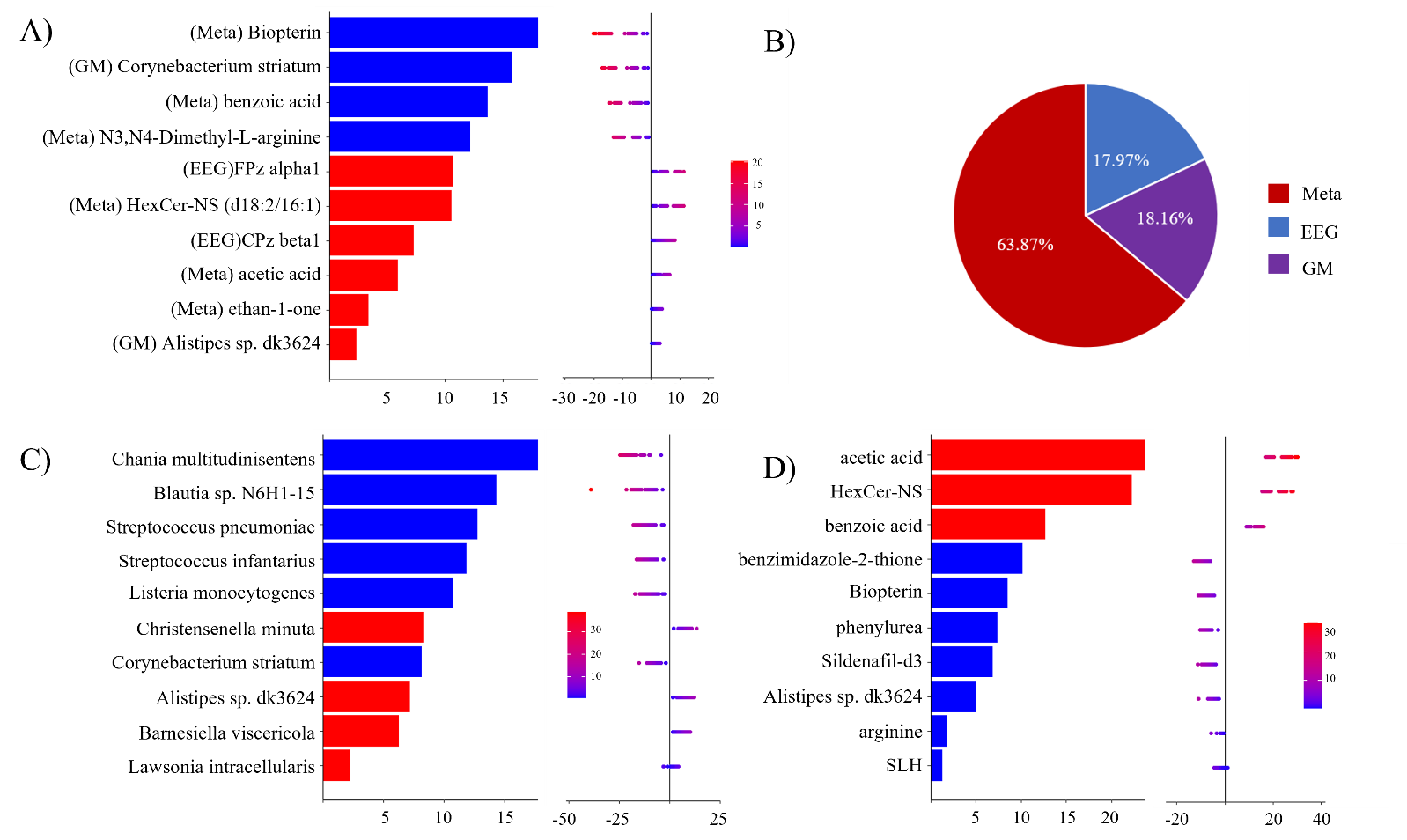


**Supplementary Figure S13.** The selected features. Panel A) The left is bar plot of selected features and their contributions of EEG, metabolomic and gut microbiota model (weight in SVM) in descending order. The right is a bee swarm plot in which each point represents a participant (n = 41). Panel B) Feature category contribution calculated by summing the weight in EEG, metabolomic and gut microbiota model. Panel C and panel D represented the gut microbiota and metabolomics respectively. In bar plot, blue bar represented higher value of the feature for association with NCDs while the red bar presented higher association with normal aging. In bee swarm plot, color indicates the value of the feature, with red higher and blue lower. Negative contribution indicates the feature attribution for prediction of NCDs while Positive contribution indicates the feature attribution of normal aging. Meta, metabolomic; GM, gut microbiota.


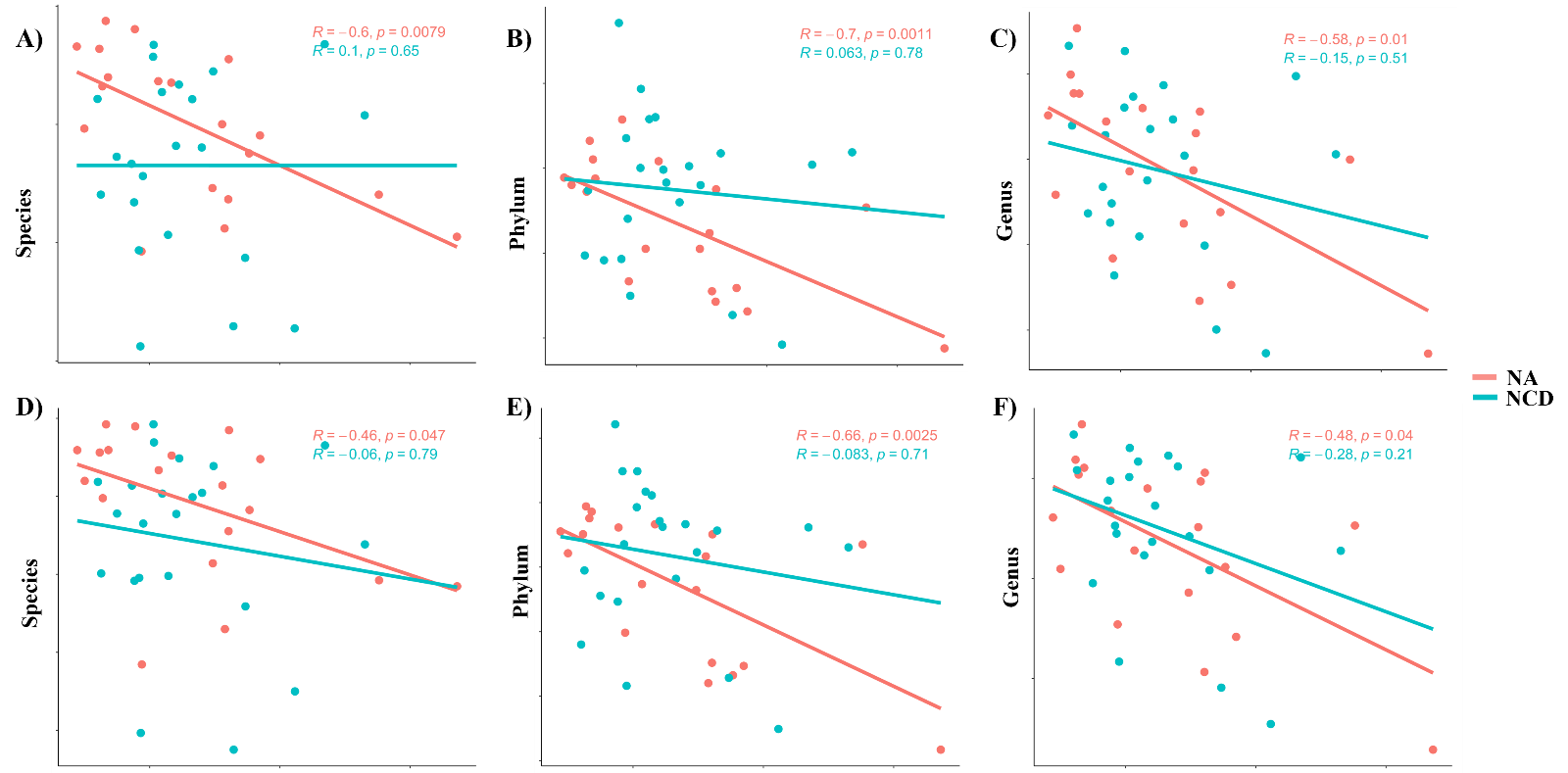


**Supplementary Figure S14.** The correlation results between EEG alpha-1 power and the diversity of gut microbiota in both NA and NCDs. Panel A-C) The Shannon index in Species, Phylum, and Genus level. Panel D-F) Simpson index in Species, Phylum, and Genus level.
